# A diffusion-based integrative approach for culturing previously uncultured bacteria from marine sediments

**DOI:** 10.1007/s42995-024-00240-2

**Published:** 2024-08-12

**Authors:** Tariq Ahmad, Sidra Erum Ishaq, Lewen Liang, Ruize Xie, Yinzhao Wang, Fengping Wang

**Affiliations:** 1 State Key Laboratory of Microbial Metabolism, School of Life Sciences and Biotechnology, Shanghai Jiao Tong University, Shanghai, 200240 China; 2 Key Laboratory of Polar Ecosystem and Climate Change, Ministry of Education, School of Oceanography, Shanghai Jiao Tong University, Shanghai, 200240 China

**Keywords:** Uncultured bacteria, Cultivation, Microbial aquarium, Low nutrient media, Recalcitrant organic substrates

## Abstract

Traditional cultivation methods with defined growth media can only isolate and cultivate a small number of microbes. However, much higher microbial diversity has been detected by cultivation-independent tools from a range of natural ecosystems. These represent a large unexplored pool of potentially novel taxa. In this study, a diffusion-based integrative cultivation approach (DICA) was developed to efficiently isolate novel taxonomic candidates from marine sediment. DICA combined a newly designed diffusion-based apparatus called a “microbial aquarium” with modified low-nutrient media. To determine the efficiency of DICA, cultivation results were compared with traditional cultivation approach (TCA). Both cultivation approaches resulted in the isolation of numerous representatives from the phyla *Pseudomonadota*, *Actinomycetota*, *Bacteroidota*, and *Bacillota*. However, the newly developed DICA also led to the successful cultivation of species from rarely cultivated phyla such as *Verrucomicrobiota* and *Balneolota.* Based on 16S rRNA analyses, the application of DICA resulted in the successful cultivation of 115 previously uncultured taxa out of a total of 196 isolates. Among these, 39 were identified at the genus level and 4 at the family level, showcasing a novelty ratio of 58%. Conversely, the TCA cultivated 12% (20/165) of novel isolates, with all at species level only. The isolated microbial diversity showed that species recovered by DICA belong to 12 different classes, twice the number produced by TCA. Overall, these results demonstrate that the newly designed DICA produces a high recovery of diverse and previously uncultured bacteria.

**Supplementary Information:**

The online version contains supplementary material available at 10.1007/s42995-024-00240-2.

## Introduction

Marine ecosystems are rich reservoirs of diverse microbial communities with an estimated abundance of 10^3^–10^10^ microbial cells/cm^3^ in sediment and 10^4^–10^7^ cells/mL in seawater. Together they comprise a major proportion of the global microbial biomass (Wang et al. 2021). In deep-sea sediments, microorganisms, including aerobic bacteria, play important roles in nutrient cycling, macromolecules degradation, and other ecological processes (Orcutt et al. 2013; Zhao et al. 2020). However, most (> 99%) of them have never been cultured and characterized under laboratory conditions (Hofer 2018). Although culture-independent studies have revealed the immense diversity and metabolic potentials of marine microbial populations, obtaining pure cultured strains remains important for a practical understanding of their morphology, physiology, and ecological functions in complex environmental processes (Lewis et al. 2021). Currently, only a small fraction of marine bacteria are available as cultures (Baker et al. 2021). There are multiple reasons to explain our inability to isolate and grow marine bacteria under laboratory conditions (Overmann et al. 2017; Stewart 2012), but the failure to maintain a natural habitat and insufficient knowledge of nutrient media that best mimic the natural growth conditions of native microorganisms are among the major concerns.

A lack of success in maintaining a natural growth environment, including access to appropriate nutrients, has caused microbe–microbe interactions that occur via signaling molecules such as peptides, siderophores, and quinones, to be disabled (D'Onofrio et al. 2010; Jung et al. 2021b). To overcome this obstacle, several in situ cultivation methods, which have tried to better reflect the natural growth conditions, have been developed and applied to a range of habitats, including sediment (Kaeberlein et al. 2002), soil (Chaudhary et al. 2019) and sponges (Jung et al. 2021c). These methods include the encapsulation of inoculum in a semi-permeable double-layer diffusion chamber (Kaeberlein et al. 2002), the use of ichip, a high-throughput cultivation device composed of several hundred mini-diffusion chambers (Nichols et al. 2010), and a diffusion bioreactor (Chaudhary et al. 2019). Each of these techniques has been useful for the cultivation of previously uncultured bacteria. Secondly, the choice of growth medium for the enrichment and isolation of microbial species is also of prime importance. Peptone, yeast extract, and simple organic substrates such as glucose, starch, pyruvate, and casamino acids are used routinely, either as an individual source or as a mixture with the underlying assumption that most microbes can likely utilize these components (Cui et al. 2016). However, these nutrient-rich media and the addition of simple organic compounds into the enrichment medium commonly yield lower biodiversity with only a few dominant fast-growing strains dominating. In low-nutrient media with more complex organic sources lead to significantly higher biodiversity (Wu et al. 2020). In deep-sea sediments, dissolved organic matter is mainly composed of recalcitrant carbon compounds and hence contributes to the available carbon source for the growth of a diverse range of microorganisms in their natural habitat (Chen et al. 2023; Wu et al. 2018). For example, previous studies have shown that media containing recalcitrant organic substrates (lignin) improved the enrichment of uncultured sedimentary *Bathyarchaeota*-8 subgroup clade (Yu et al. 2018, 2023).

In recent years, several well-designed cultivation techniques have been developed to culture uncultured bacterial strains. These techniques include extending the enrichment/incubation period to support slow-growing and low-abundance microbes (Hu et al. 2021; Liang et al. 2022; Lv et al. 2022; Pulschen et al. 2017). Moreover, addition of signaling molecules in growth media and co-culturing with helper organisms have been employed to indicate the presence of an appropriate growth environment (Knobloch et al. 2019; Rygaard et al. 2017). Dilution and physical separation of cells have been utilized to decrease competition (Stingl et al. 2007). Furthermore, alternative gelling agents and modifications to the preparation of agar media have been implemented to minimize oxidative stress (Kato et al. 2018). All of the above-mentioned techniques have allowed the isolation of many previously uncultured microbes. However, these methods are not sufficient to mitigate the known difficulties in isolating the majority of microbes, particularly from hard-to-culture groups i.e., *Acidobacteria* and *Verrucomicrobiota*. These groups have been widely reported from a range of ecosystems, often in high abundance, but very few of them have been cultured (Solden et al. 2016). Hence, there is still a huge gap for improvement and an urgent need to develop more efficient, easily reproducible, and practical approaches that can best mimic natural growth conditions. In this study, a diffusion-based integrative cultivation approach for uncultured marine sediment bacteria was developed that allows for the growth of marine bacteria in their natural habitat. The newly developed approach incorporates a diffusion-based device called “microbial aquarium”, coupled with modified enrichment media to mimic the natural setting of marine bacteria. The findings of this study contribute to the development of cultivation techniques used for previously uncultivable marine bacteria, enabling their recovery from marine sediment and cultivation in the laboratory setting.

## Materials and methods

### Sample collection

Sediment samples were collected from the South China Sea (S1) and Mariana Trench (S2) during the cruises SONNE-269 in 2019 and TS21-2 in 2021, respectively. The sediment samples (S1 and S2) were collected using a multi-corer at a water depth of 1896.9 m and 9010.3 m (18.81°N, 115.83°E and 142.34°E, 11.11°N). Once retrieved on board, samples were immediately placed into sterile Nasco sampling bags under aerobic conditions and stored at 4 ℃ until experimentation.

### Nutrient media

For the cultivation of marine bacteria, the following three types of modified nutrient media i. 0.5% alkali-lignin (Lig-medium), ii. 0.5% starch (St-medium), and iii. artificial seawater (ASW-medium) were formulated. The prior two media were mixed with artificial seawater (g/L): 26.0 g NaCl, 5.0 g MgCl_2_ · 6H_2_O, 1.4 g CaCl_2_ · 2H_2_O, 4.0 g Na_2_SO_4_, 0.3 g NH_4_Cl, 0.1 g KH_2_PO_4_, 0.5 g KCl, 1.0 mL trace element mixture, 30.0 mL1 mol/L NaHCO_3_ solution, 1.0 mLvitamin mixture, 1.0 mL thiamine solution, and 1.0 mL vitamin B_12_ solution. These three modified media were used for the initial enrichment of the sediment samples, while 50% diluted marine 2216E and R2A agar media were used for later sub-cultivation on 1.5% agar plates. The composition of each medium is described in Table S5.

### Design of the microbial aquarium and experimental setup for diffusion-based integrative culturing

**Fig. 1:**
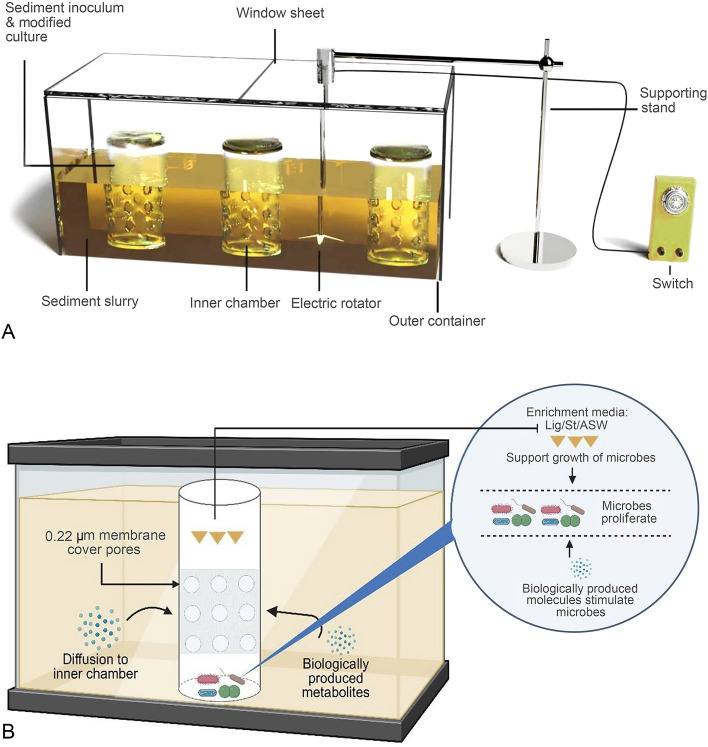
Design, working principle, and structure of cultivation device. **A** Schematic diagram showing the overall experimental setup of apparatus “Microbial aquarium” (MA). **B** Working principle of DICA

In this study, the “microbial aquarium” apparatus used is based on diffusion phenomena and consists of a rectangular glass box as an outer chamber (30 L, 50 cm × 30 cm × 20 cm, width × height × depth) with three inner, semi-permeable cylindrical glass chambers (each chamber is a 2 L glass container, 10 cm × 24 cm × 8 cm, width × height × depth) (Fig. 1A). A total of 15 holes (6 mm in diameter) were drilled over the surface of each inner chamber. These holes were then covered with a 0.22 µm pore size polycarbonate membrane filter paper (PR04769, Merck Millipore, Ireland), firmly attached using glue (08d-2, Contact CR glue, China great wall industry, Shanghai, China) (Fig. 1B). The inner chambers were placed inside the outer container and the gap between inner and outer chambers walls was filled with a 0.5% (w/v) sediment slurry from same samples (Fig. S1). Before the addition of sample and nutrient media, the newly designed apparatus was sterilized with 75% (v/v) ethanol, rinsed with particle-free molecular grade water followed by drying under UV-light (TUV 8W/G8 T5, Philips, Wrocław, Poland) in a laminar flood hood for 12 h. After sterilization, 0.25 g of sediment, along with 500 mL of Lig-, St-, and ASW media, were added to the three inner chambers; the outer chamber was filled with 75 g of sediment mixed with 15 L of ASW. The openings of the inner chambers were tightly covered with glass lids while the outer chamber was closed with a glass cover sheet (Fig. S1). This whole apparatus was kept at 25 °C for 4 weeks. To effectively stir and homogenize the sample, an electric rotator was used in the outer container (Fig. 1A), while a sterile 25 mL pipette was used manually in the inner chambers at 72 h intervals. To prevent airborne microbial contamination, the apparatus was taken, opened, and stirred in a UV-sterilized laminar flow hood. After 4 weeks of enrichment, a 1 mL sample was taken from each inner chamber and was serially diluted, as mentioned below for the traditional cultivation approach. 70 µL of aliquot from 10^–4^, 10^–5^, 10^–6^, and 10^–7^ dilutions were taken in duplicate and inoculated onto freshly prepared, 50% diluted marine 2216E and R2A agar plates. These inoculated plates were then incubated at 25 °C for several weeks (until new colonies appeared) aerobically. For comparison of DICA and TCA, the above-mentioned sub-cultivation steps were equally followed for both methods.

### Traditional cultivation approach

**Fig. 2:**
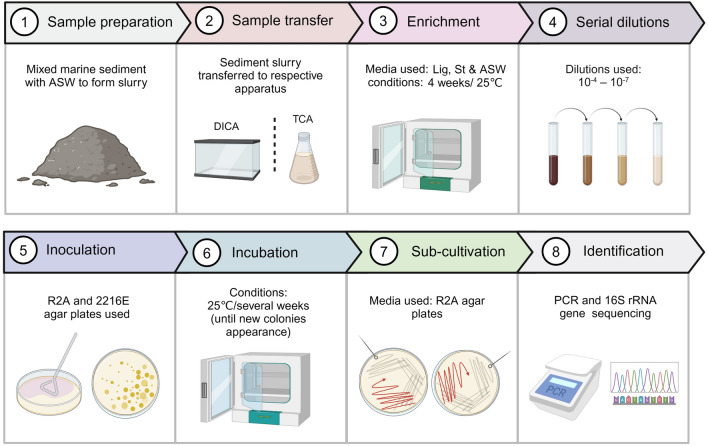
Workflow of the experimental steps followed for cultivation of bacterial species

For comparative analysis, deep-sea sediment bacteria were also cultured using a TCA (Fig. 2). A 0.25 g sediment sample was added to a conical flask containing 500 mL of medium, (0.5% Lig-, St- and ASW-media, respectively). All of the conical flasks were kept in a 25 °C incubator for 4 weeks. The sample in each flask was mixed manually at 72 h intervals, following a similar pattern to that for the inner chambers. After 4 weeks of enrichment, 1 mL of the sample was separated from each flask and was serially diluted from 10^–1^ to 10^–7^ with sterile ASW. A 70 µL of aliquot from each of the 10^–4^, 10^–5^, 10^–6^, and 10^–7^ dilutions was taken in duplicate and spread on agar plates. These plates were then incubated aerobically at 25 °C for several weeks. Colonies that appeared on the agar plates during the incubation period were picked and purified on 50% diluted marine R2A agar plates, not including obvious duplicates. Colonies that had the same color, shape, size, and growth time as another in the similar type of medium were considered duplicates, and removed to avoid unnecessary repetition. To ensure accurate observations, a magnifier was used to assess the color, shape, and size of the colonies. After successful purification of the colonies, PCR, 16S rRNA gene sequencing, and analysis were performed (Fig. 2).

### Identification of isolates based on 16S rRNA gene sequencing

Taxonomic identification was performed by sequencing full-length 16S rRNA gene sequences. The colony material was used directly as a template for PCR. The 16S rRNA gene was amplified using the universal primers 27F (5`-AGAGTTTGATCCTGGCTCAG-3`) and 1492R (5`-GGTTACCTTGTTACGACTT-3`) with the following cycling conditions: initial denaturation at 95 °C for 5 min, followed by 30 cycles of 95 °C for 30 s, 57 °C for 1 min, and 72 °C for 1 min 30 s, with a final step at 72 °C for 10 min (Woodman et al. 2008; Heuer et al. 1997). The PCR products were sequenced commercially by fluorescent dye terminator sanger sequencing (Shanghai Sunny Biotech Co., Ltd, Shanghai, China). To compare the closest phylogenetic relatives of isolates, all of the sequences were compared using NCBI-BLAST (Altschul et al. 1990) and EzBioCloud server (Yoon et al. 2017). The 16S rRNA sequences were retrieved from the NCBI nr database and aligned using the stand-alone version of the MAFFT alignment tool with the parameters “–localpair,–maxiterate 1000” (Katoh and Standley 2013). The resulting alignment was then filtered by TrimAL (Capella-Gutiérrez et al. 2009) with the “automated1” option. The phylogenetic analysis was performed via IQ-tree under the GTR + F + R7 model and 1000 ultrafast bootstraps (Nguyen et al. 2015).

### DNA extraction and amplicon sequencing targeting the 16S rRNA gene

To compare the recovered bacterial diversity with the microbial molecular signatures in the studied samples, amplicon sequencing on 16S rRNA genes was performed. Genomic DNA was extracted from (0.5 g) sediment samples using a DNeasy Power Soil kit (Qiagen, Hilden, Germany), according to the manufacturer’s protocols. The DNA was extracted in triplicate. The purity and concentration of the DNA samples were determined using a NanoDrop One (Thermo Fisher Scientific, MA, USA). The extracted sediment DNA was used to amplify the 16S rRNA gene using the amplicon forward primer 515F (5′-GTGCCAGCMGCCGCGGTAA-3′) and reverse primer 806R (5′-GGACTACHVGGGTWTCTAAT-3′) (Zeng and An 2021), with PCR reactions, containing 25 μL 2 × Premix Taq (Takara Biotechnology, Dalian Co. Ltd., China), 1 μL of each primer(10 μmol/L) and 3 μL of DNA (20 ng/µL) to a volume of 50 µL, these were amplified by thermocycling: 5 min at 94 °C for initialization; 30 cycles of 30 s denaturation at 94 °C, 30 s annealing at 52 °C, and 30 s extension at 72 °C; followed by 10 min final elongation at 72 °C. The length and concentration of the PCR products were detected by 1% agarose gel electrophoresis and purified with E.Z.N.A. Gel Extraction. Sequencing libraries were generated using NEBNext^®^ Ultra^™^ II DNA Library Prep Kit for Illumina (New England Biolabs, MA, USA) following the manufacturer's recommendations, and index codes were added. At last, the library was sequenced on an Illumina Hiseq platform and 250 bp paired-end reads were generated (Guangdong Magigene Biotechnology Co., Ltd. Guangzhou, China). Sequence data were analyzed with QIIME 2 (v.2020.11) (Estaki et al. 2020).

## Results

### Cultivations setup

In this study, the diffusion-based integrative cultivation approach was established compared with conventional methods of isolation of aerobic bacteria, as described in the Materials and Methods. The schematic diagram (Fig. 1A), working principle (Fig. 1B), and structure (Supplementary Fig. S1) of the diffusion-based cultivation device called “microbial aquarium” are shown in Fig. 1. This apparatus is based on the concept of in situ cultivation with the ability to enable the exchange of small molecules between two chambers, particularly from regions of high concentration to regions of low concentration. Here, a concentration gradient was induced by adding a large volume (15 L) of 0.5% sediment slurry to the outer container, which would have more likely had a high concentration of unknown essential nutrients/signaling molecules compared to each of the inner chambers, which contained (0.5 L) of 0.05% sediment slurry. The concentration gradient of the resulting sediment slurry of 1:10 between the inner and outer chambers facilitated the diffusion of these molecules from a region of high concentration (outer container) to a region of low concentration (inner chambers). This minimizes the chemical differences on either side of the inner chambers and mimics the natural sediment environment. The application of DICA enabled the availability of substrate complexes to the bacteria residing in the inner chambers, which may stimulate and improve the growth of a diverse range of bacterial species. The substrate complexes included the initially added organic/low-nutrient media to the inner chambers and the naturally produced essential growth factors that diffused through the membrane. The workflow of the experimental steps followed in the cultivation experiments is highlighted in Fig. 2.

### Taxonomic analysis of bacterial diversity in deep-sea sediment samples

Culture-independent and dependent analyses were performed on the two deep-sea sediment samples used in the study (S1 and S2). Culture-independent analysis revealed the presence of high bacterial diversity in both samples (Supplementary Table S1 and Table S2). The 16S rRNA gene sequences obtained through Illumina HiSeq sequencing of initial-unamended samples showed affiliations with seven major phyla, namely *Pseudomonadota, Bacteroidota, Planctomycetota, Desulfobacteria, Candidatus* Hydrogenedentes*, Verrucomicrobiota,* and *Bacillota,* which all had a relative abundance exceeding 0.1%. Additionally, 22 minor phyla were detected, each with a relative abundance below 0.1% (Table S1a, S1b). The culture-independent analysis identified approximately 177 major genera each with a relative abundance higher than 0.01% in the initial unamended samples. Furthermore, the 16S rRNA gene sequence analysis of the samples incubated for one month showed that the samples enriched by DICA had higher bacterial diversity compared to the TCA enriched samples (Table S1a, S1b). In addition, several archaeal groups, including *Nanoarchaeota*, *Crenarchaeota*, *Euryarchaeota*, and *Thaumarchaeota*, were also observed during the V3–V5 Illumina amplicon sequencing of the samples. However, the percentage of archaea in the entire microbiome was very low, (0.005–0.1%) and as our primary focus was on bacterial isolation, data from the archaeal groups were not included.

**Fig. 3:**
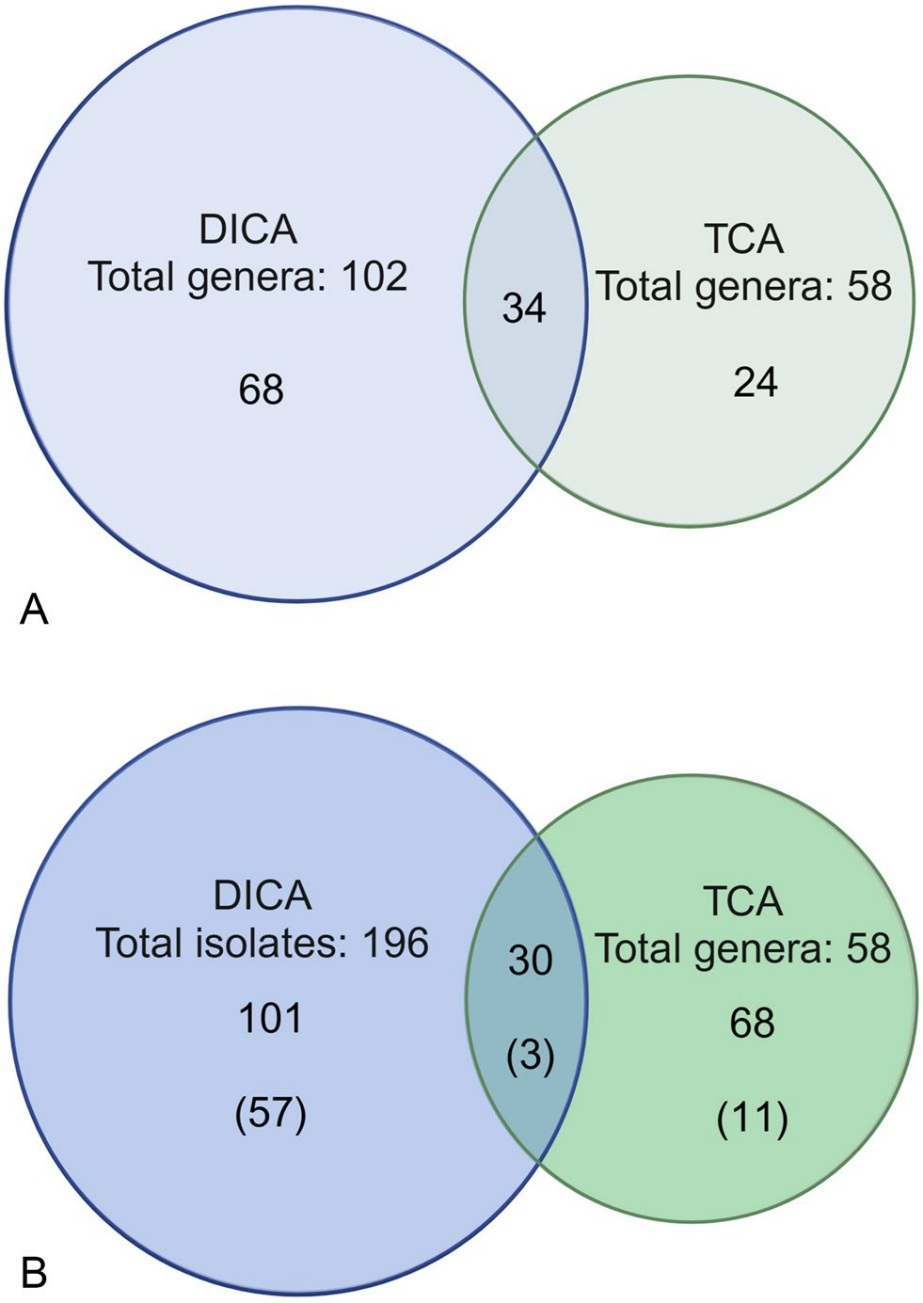
Overlap among culture collections obtained using DICA and TCA at genus **A** and species level **B**. Values in the center of each circle represent the individual number of genera **A** and species **B** isolated by each method; values in the overlapping areas represent the numbers of co-isolated genera/species. The numbers in parentheses show the numbers of individual novel species in Fig. 3B. Venn diagrams were made with Venn diagram Plotter-venny 2.1. (Oliveros 2007–2015)

A total of 361 pure bacterial strains were isolated from samples S1 (184) and S2 (177) (Table S2a, S2b), which belonged to four major phyla (*Pseudomonadota, Bacteroidota, Verrucomicrobiota,* and *Bacillota)* and three minor phyla (*Actinomycetota, Cyanobacteria,* and *Balneolota*). The isolates obtained from both cultivation methods belonged to 126 genera, of which 68 were exclusively isolated by DICA, 34 were obtained by both DICA and TCA and 24 were isolated by TCA only (Fig. 3A). The DICA method enabled the isolation of 196 species from 12 class level taxonomic groups whereas TCA enabled isolation of 165 species from only six taxonomic classes. The DICA yielded 101 individual species that were absent from the TCA collection, with an overlap of 30 species (Fig. 3B).

### Comparison of total novel strains recovered by DICA and TCA

**Fig. 4:**
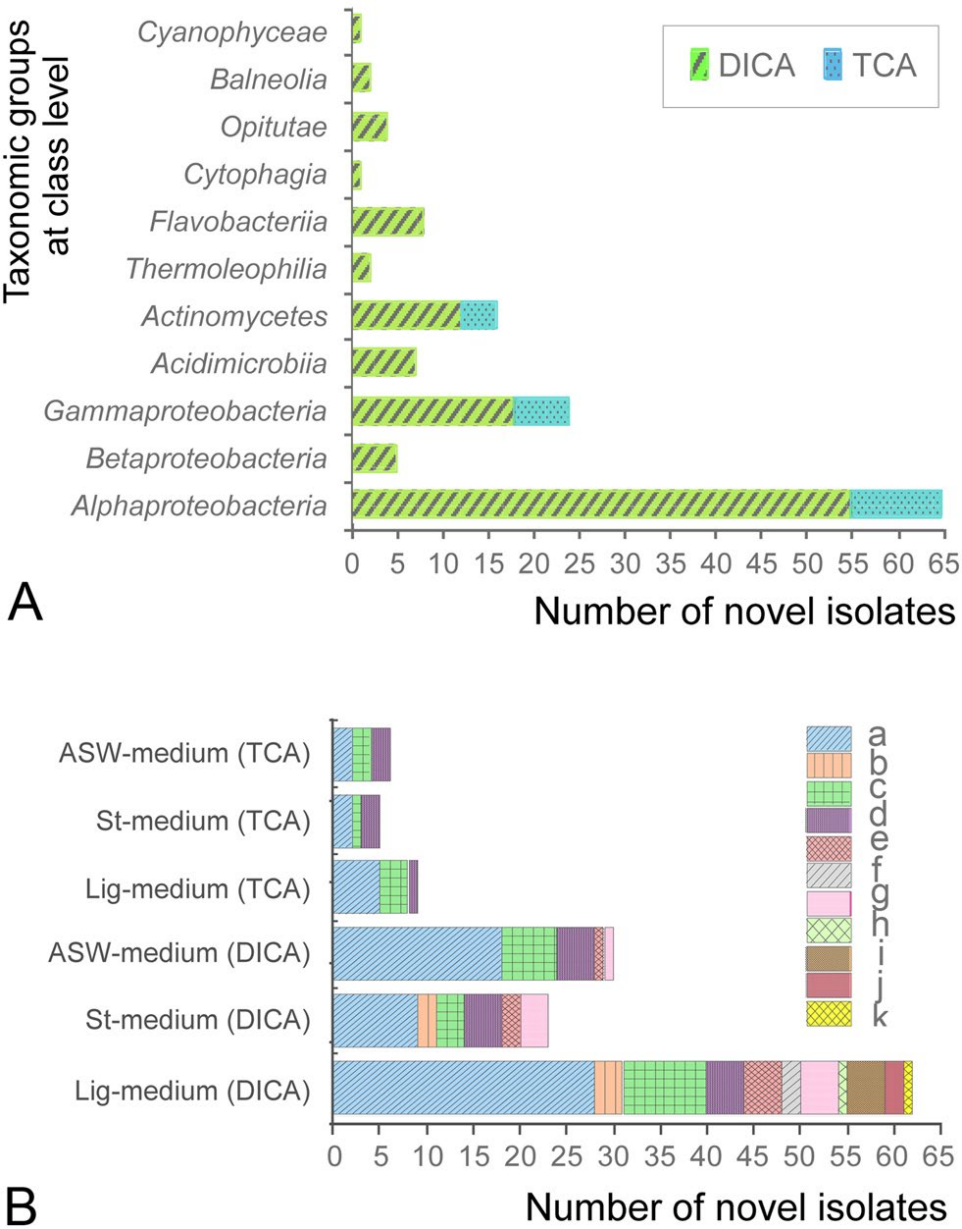
Comparison and affiliation (at class level) of total putative novel isolates recovered. **A** Number of novel isolates obtained by DICA vs TCA. **B** Number of novel isolates obtained by different modified nutrient media along with cultivation approaches. a represents class *Alpha-proteobacteria,* b *Beta-proteobacteria,* c *Gamma-proteobacteria,* d *Actinomycetes,* e *Acidimicrobiia,* f *Thermoleophilia,* g *Flavobacteriia,* h *Cytophagia,* i *Opitutae,* j *Balneolia,* and k *Cyanophyceae*

Isolates with a 16S rRNA gene sequence similarity below the threshold value of 98.65% were considered to be novel species (Kim et al. 2014; Nguyen et al. 2018). Of all the bacteria present in the studied sediment samples, the diffusion-based integrative technique enabled the successful cultivation of 115 putative novel species from 11 taxonomic classes, affiliated with six phyla, whereas TCA recovered 20 novel strains from only three classes of two phyla (Table S2a). Interestingly, 58% of these novel isolates were slow-growers, as they showed growth (formation of colonies) after an incubation period of more than a week on agar plates (Kato et al. 2018). Of the 79 potential slow-growing isolates, 74 were isolated using the DICA method, as indicated in Table S2a. Furthermore, the maximum number of novel isolates obtained using both cultivation approaches were predominantly from *Alphaproteobacteria*. No representatives from the *Flavobacteriia, Acidimicrobiia, Betaproteobacteria*, *Opitutae*, *Thermoleophilia*, *Balneolia*, *Cytophagia,* or *Cyanophyceae* classes were cultured using the TCA, while they were by DICA (Fig. 4A).

### Comparison of total novel strains recovered by different nutrient media

The significance of low-nutrient and organic substrates containing media for isolating novel bacteria from diverse taxonomic groups was also evaluated. The results described in Fig. 4B show that nutrient medium composed of labile organic substrate (starch) was not effective in yielding novel bacteria from sediment, compared to medium composed of recalcitrant organic substrate (lignin) and low-nutrients (ASW). Of the three modified media tested, Lig-medium yielded the most abundant and diverse range of novel bacterial species from both techniques, particularly from DICA, as compared to St and ASW-modified media. Out of the 135 novel strains, 71 were obtained from Lig-modified medium, 37 from ASW- and 27 from St-modified media.

**Fig. 5:**
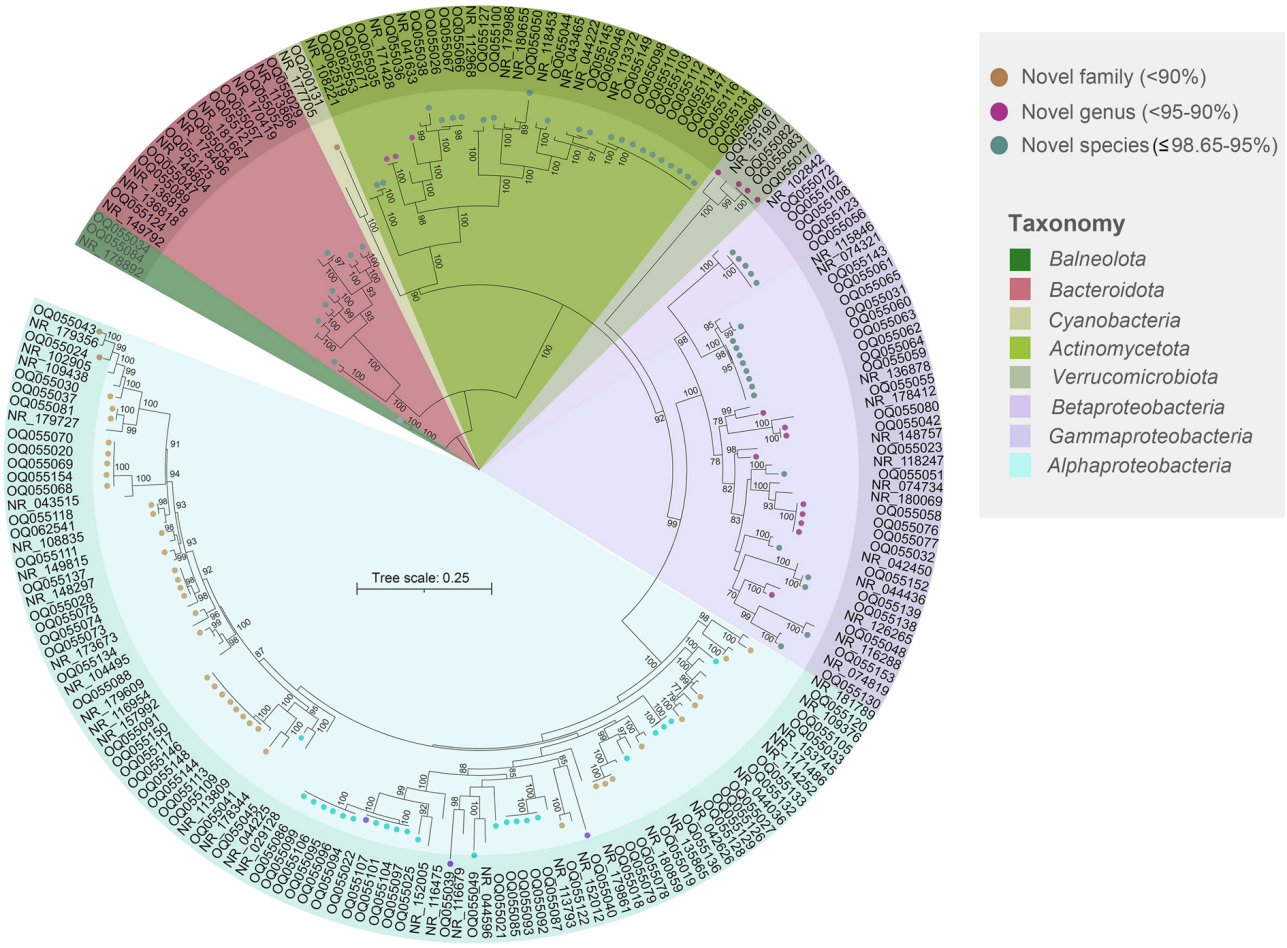
Phylogenetic tree of 135 representative putative novel bacterial isolates based on nearly full-length 16S rRNA gene sequencing. Bootstrap values of ≥ 50% are shown in the tree. Accession numbers for 16S rRNA sequences of potential novel bacterial isolates and their closely related known bacteria (as references) are shown in the tree. Novel candidate species (purple), novel candidate genus (golden), and novel candidate family (green) are shown by colored dots

Based on the comparison of the 16S rRNA gene sequence similarity percentages, 135 novel bacterial isolates could be assigned to putative novel taxa at family (< 90%), genus (< 95–90%), and species level (≤ 98.65–95%) (Jung et al. 2021b). Of these potential novel isolates, 92 were of new species, of which 72 were isolated by DICA and 20 by TCA, similarly 39 isolates were identified at the genus level and four at the family level as new candidates; these were only recovered by DICA (Fig. 5). The newly recovered isolates belong to 11 different class level groups, affiliated with six phyla. These groups include the classes *Alpha*, *Beta,* and *Gammaproteobacteria* from the phylum *Pseudomonadota*, the classes *Actinomycetes*, *Acidimicrobiia,* and *Thermoleophilia* from the phylum *Actinomycetota* and the classes *Flavobacteriia* and *Cytophagia* from the *Bacteroidota* phylum. Furthermore, the classes *Opitutae, Balneolia,* and *Cyanophyceae* were associated with the phyla *Verrucomicrobiota, Balneolota,* and *Cyanobacteria*, respectively*.* Of all these taxonomic groups, TCA enabled isolation from *Alphaproteobacteria, Gammaproteobacteria,* and *Actinomycetes* only*,* whereas DICA successfully recovered species from all the 11 groups mentioned (Supplementary Table S3). Overall, the taxonomic analysis showed the recovery of 115 novel strains from DICA, while the use of TCA resulted in the acquisition of 20 novel isolates (Table S2a). Among these 135 novel isolates, 68 showed close affiliation with uncultured environmental species as compared to cultured species and were considered to be previously uncultivated bacteria. Almost 90% of these previously uncultured bacteria were isolated by DICA (Table S2a).

## Discussion

Most (> 99%) bacteria identified by culture-independent surveys in various natural environmental samples, particularly those from marine habitats, remain uncultivated (Hofer 2018). These uncultured taxa are regarded as a significant source of natural bio-products and key players in biogeochemical cycles (Vartoukian et al. 2010; York 2018). Therefore, there is a compelling need to apply a greater effort to develop alternative cultivation methods for the isolation and culture of these uncultivated bacteria to improve the bio-prospecting of marine microbial resources. In this study, the DICA method was applied to isolate novel and uncultured strains from marine sediment. The DICA method includes a newly designed apparatus, the “microbial aquarium”, which is based on the diffusion principle with the integration of recalcitrant/labile organic and low-nutrient sources. The utilization of DICA facilitated the cultivation of numerous previously uncultured bacteria, including slow-growing types. This was made possible by the provision of two essential factors simultaneously. Firstly, the DICA provided access to naturally produced growth factors, which play a vital role in stimulating bacterial growth (D'Onofrio et al. 2010). Secondly, the availability of a more natural growth medium further enhanced the growth of these bacteria (Chen et al. 2023). Additionally, the implementation of an extended incubation period played a supplementary role in enabling the successful cultivation of previously uncultured bacteria, particularly of the slow-growers (Kato et al. 2018).

### Novelty and advantage of the DICA

The DICA method applied here is distinct from previously reported in situ cultivation studies in regard to the structure of the apparatus, the membrane pore size, and the use of modified enrichment media. The in situ cultivation method of Kaeberlein et al. (2002), used a diffusion chamber composed of a stainless-steel washer covered with 0.03 µm pore size membranes along with an agar matrix as a solid nutrient medium (Kaeberlein et al. 2002). Later, Nichols et al. (2010) designed a high-throughput in situ cultivation device called “ichip”, which used an array of several hundred small diffusion chambers with a 0.03 µm pore size membranes located on top and bottom, of the chambers, which were loaded with a suspension of microbial cells in warm agar (Nichols et al. 2010). A recently developed in situ cultivation method, used a diffusion bioreactor composed of two plastic containers and 0.4 µm pore size membranes, along with conventional enrichment nutrient media (Chaudhary et al. 2019).

In this study, a newly designed diffusion-based apparatus was used. The use of a large outer container provided the capacity for a greater amount of natural sediment, which most probably increased the concentration of unknown essential nutrients. Furthermore, the use of an appropriate pore-size membrane, along with a small electric rotator and a sterile pipette as sample mixers, significantly enhanced the probability of diffusion of growth factors from regions of high concentration (outer chamber) to those of low concentration (inner chambers). This strategic setup also facilitated the dispersion of nutrients, oxygen, and microbes throughout the sample, thereby improving the replication of natural sediment systems. Similarly, previous studies have reported that large amounts of natural samples on the outer side of an inner chamber (Chaudhary et al. 2019), membranes with pore sizes of 0.03 to 0.4 µm (Crump and Richardson 1985), and proper mixing of samples (Bollmann et al. 2007); can enhance the diffusion and circulation of growth factors. This enhancement subsequently paves the way for the proliferation of a diverse range of microbial species.

During the sub-cultivation and isolation of pure colonies, the inoculation of the diluted sediment samples onto agar plates and incubation for an extended period allowed sufficient time for the bacteria, including slow-growers to develop and proliferate. The study by Kaeberlein et al. (2002) found that subjecting some environmental microorganisms to one or more cycles of incubation in a diffusion-based in situ cultivation device, promoted their sub-cultivation on standard agar plates in vitro. Subsequently, Bollmann et al. (2007) confirmed this method and suggested two potential explanations. Firstly, the representatives of such isolates were too few at the start of the experiment and needed to be enriched within the diffusion chamber before appearing on the agar plates in sufficient numbers for effective isolation. Secondly, the successful adaptation of these isolates to grow on agar plates needed prior growth events within the simulated natural environment of the diffusion chamber. Both these characteristics may have contributed to a controlled and sustained supply of essential nutrients, inter-microbial interactions, or other unknown factors provided by diffusion-based in situ cultivation devices (Bollmann et al. 2007; Stewart 2012). In the experiments described here, a single round of incubation within a diffusion chamber resulted in the successful cultivation of a number of novel and diverse environmental isolates capable of thriving in a laboratory setting on agar plates.

The advantage of this apparatus lies in its ability to provide an external supply of growth factors and nutrients while simultaneously allowing microbes within the inner chambers to grow in a less competitive environment due to the use of a diluted inoculum (Nichols et al. 2010). Conversely, in a conical flask, microbes lack the ready influx of growth factors. Nevertheless, to increase the concentration of growth factors in the conical flask, a couple of approaches should be considered. One option is to add more sediment, but this may inadvertently amplify the prevalence of dominant microbes, thereby making the competitive environment more pronounced (Wu et al. 2018). An alternative solution involves incorporating sediment extract, which may also be effective (Nguyen et al. 2018). However, this introduces complex, resource-intensive, and time-consuming steps into the process, potentially impeding the overall workflow.

### Efficiency of the DICA over TCA in isolating diverse and novel strains

For comparison, all of the cultivation conditions and nutrient media used were similar for both the newly designed and traditional cultivation approaches (Fig. 2). Comparing the bacterial composition of initial-unamended sediment samples observed by Illumina HiSeq sequencing analysis with that of the enriched samples, it was found that the samples enriched by DICA, had a high bacterial diversity in comparison to the TCA enriched samples (Table S1a, S1b). This suggests that mimicking the in situ growth conditions by enabling access to biologically produced growth factors and native nutrient media simultaneously to microbial communities, can lead to an increase in bacterial diversity compared to traditional approaches (Kaeberlein et al. 2002; Wu et al. 2018). The DICA method led to the isolation of several previously uncultured bacteria, that were present in the environmental samples but were not cultivated by TCA (Table S4). A total of 58 and 102 genera were present in the cultures isolated by the TCA and DICA methods, respectively (Fig. 3A). This shows that isolates obtained by DICA recovered a higher diversity from the samples compared to the TCA. It was also noted that *Planctomycetota, Desulfobacteria,* and *Candidatus* Hydrogenedentes were not isolated, even though a high proportion of these groups were found in the deep-sea sediments (Table S1). These bacteria may have some specific growth preferences, such as N-acetylglucosamine supplemented with antibiotics (Lage and Bondoso 2012) and anaerobic conditions, respectively (Kuever et al. 2014), which were not met in this study.

The 16S rRNA gene sequencing analysis showed that the DICA method yielded novel isolates in much higher numbers and levels than TCA. DICA recovered 115 putative novel isolates out of 196, including 72 at the species level, 39 at the genus level, and 4 at the family level, showing 58% novelty efficiency compared to 12% (20/165) for the TCA. This allowed the isolation of 20 potential novel candidates at species level only (Table S2a). The recovery of several novel isolates by TCA indicates that a small fraction of the largely unexplored microbial population, may not require special growth conditions and can still be successfully cultivated using conventional methods (Jung et al. 2021a; Momper et al. 2018). In addition, the DICA method led to the isolation of strains from an additional six diverse classes that were not obtained by TCA. Almost 77% of species (24 out of 31) belonging to these taxa were novel taxonomic candidates (Table S2a). This result suggests that strains belonging to these groups are non-conducive to cultivation with the TCA and that the growth conditions provided by the DICA are essential to cultivate such bacteria. In DICA, the integration of a microbial aquarium with modified enrichment media improved the simulation of a natural growth environment for inoculated microbes and thus successfully increased the recovery of previously uncultured taxa. However, the traditional use of a conical flask in TCA resulted in a consistent level of bacterial isolation with a very low significant difference between the three modified nutrient media used. These results demonstrate that the isolation of novel/previously uncultured bacteria is affected by the cultivation equipment as well as nutrient sources and media, as marine bacterial communities are nutritionally dependent and are influenced by the origin and concentration of nutrients (Sherr and Sherr 2008).

### Effects of enrichment media on isolation of diverse and novel strains

A second important strategy for meeting the needs of uncultivated bacteria is to provide them with a growth medium that is most similar to their natural nutrient sources and can support the growth of bacteria belonging to various groups. In the present study, three kinds of modified enrichment media were evaluated. The results show that the highest recovery of diverse and novel bacterial strains occurred using Lig- medium as compared to St- medium. This indicates that more labile organic substances commonly lead to a biased selection of fast-growing bacteria from a few dominant groups (Wawrik et al. 2005). However, recalcitrant organic substrates, being more natural to marine sediment (Wu et al. 2018), are less labile and promote the growth of a more diverse and distinct group of bacteria, including slow-growing and rare taxa (Wu et al. 2020). In addition, the ASW-modified medium, which contains no organic compounds, was also observed to be promising for the growth of previously uncultured deep-sea bacteria. This highlights the fact that numerous indigenous bacteria, such as members of the ubiquitous SAR11 clade, prefer seawater and oligotrophic growth media (Rappé et al. 2002). Similarly, a study conducted by Henson et al. 2016, reported the isolation of several novel isolates from the Gulf of Mexico using artificial seawater media (Henson et al. 2016). The nutrient-enriched media permitted the isolation of species from only a few dominant groups and frequently failed to isolate members of previously uncultured groups (Bartelme et al. 2020). Several other studies have reported on the use of recalcitrant organic and low-nutrient sources that led to the enrichment of *Bathyarchaeota* (Hu et al. 2021) and isolation of ~ 70% of species within the *Marinilabiliales* order of the *Bacteroidetes* phylum, respectively (Mu et al. 2018).

### Taxonomy and metabolic potential of the novel isolates

A large number of uncultured bacterial species recovered in this study belong to the *Pseudomonadota* and *Actinobacteria* phyla. This is a similar outcome to that obtained by others using in situ cultivation methods (Jung et al. 2021a, 2021c). However, here, several novel and uncultured species from rarely cultivated phyla such as *Verrucomicrobiota, Balneolota,* and *Cyanobacteria* were also successfully recovered. This indicates that the newly designed diffusion-based apparatus, together with the use of modified nutrient media, effectively mimics natural growth conditions, which facilitates the growth of hard-to-culture bacterial species. Several previous studies have reported on the growth preference of these rarely cultivated groups from natural habitats, using extended incubation times and recalcitrant organic substrates (Choi et al. 2009; He et al. 2017; Pathak et al. 2018).

The unexplored microbial diversity is of great interest to both basic and applied biological sciences due to their metabolic activities described by omics-based studies (Hofer 2018; Wang et al. 2021). The novel bacterial strains recovered in this study are anticipated to have significant metabolic capabilities. For example, the genomic information of potential novel strains, coded as LMO-JJ12 (OQ055024) belonging to *Rhodobacteraceae*, LMO-JJ14 (OQ055025) and LMO-SO8 (OQ0550022), belonging to *Thalassospiraceae*, reported in our previous study (Ishaq et al. 2022), indicates that these strains can potentially be key players in the sulfur cycle, as they possess the complete sulfur oxidation (Sox) pathway. Similarly, another putative novel strain LMO-MO1 (OQ055016), a member of the *Opitutaceae* family, may be a prolific degrader of complex organic polymers, owing to their abundance of Carbohydrate Active enzymes (CAZymes) genes (Ishaq et al. 2022). Each of these four bacterial strains was isolated using the newly developed DICA (Fig. 4A). Many new isolated species that are affiliated with taxonomic sub-groups *Rhizobiales* and *Alcanivoracaceae* of class *Alpha-* and *Gamma- proteobacteria*, respectively (Fig. 4A), are expected to be important in the degradation of organic pollutants and xenobiotics, respectively, as their closest related members within these groups are well known for such activities (Schleheck et al. 2004; Zadjelovic et al.^2020^).

## Conclusion

In summary, the DICA method developed in this study, based on a diffusion system with the integration of modified nutrient media, successfully enhanced the recovery of phylogenetically novel and putative metabolically important bacterial isolates. The cultivation strategy is a promising approach to isolate marine bacteria from a diverse range of groups and represents another step forward in addressing the challenges of culturing previously uncultured bacteria. This will benefit the future development of relevant culturing techniques. Therefore, we expect this integrative approach will enable the cultivation of various phylogenetically and functionally novel bacteria from diverse environmental samples.

## Supplementary Information

Below is the link to the electronic supplementary material.

Supplementary file1 (PDF 551 KB)

## Data Availability

Newly determined nucleotide sequences data of all isolated pure strains have been deposited in NCBI GenBank under accession numbers OQ055016 to OQ055114, OQ055116 to OQ055154, OQ062519 to OQ062600, OQ071638 to OQ071697, and OQ071711 to OQ071790. The 16S rRNA gene sequencing data of two studied sediment samples were deposited in the NCBI Sequence Read Archive (SRA) under the accession numbers SRR23603883 and SRR23603884.
