# Supplementary material for: A diffusion-based integrative approach for culturing previously uncultured bacteria from marine sediments
Source: Mar Life Sci Technol. 2024 Aug 12;7(3):466–77. doi: 10.1007/s42995-024-00240-2 (PMC12413390; doi:10.1007/s42995-024-00240-2)
Supplement: Supplementary file 1 — Supplementary file1 (PDF 551 KB) [file 42995_2024_240_MOESM1_ESM.pdf]

Supplementary File – Marine Life Science and Technology (MLST)

Title: A diffusion-based integrative approach for culturing previously uncultured bacteria from marine sediments

Tariq Ahmad[1] · Sidra Erum Ishaq[1] · Lewen Liang[2] · Ruize Xie[2] · Yinzhao Wang[1] · Fengping Wang[1,2*]

Affiliations: [1] State Key Laboratory of Microbial Metabolism, School of Life Sciences and Biotechnology, Shanghai Jiao Tong University, Shanghai, 200240, PR China; [2] Key Laboratory of Polar Ecosystem and Climate Change, Ministry of Education; School of Oceanography, Shanghai Jiao Tong University, Shanghai 200240, China

Running title: A diffusion-based integrative approach towards cultivation of previously uncultured bacteria in marine sediment

Corresponding author: Professor Fengping Wang

Email address: fengpingw@sjtu.edu.cn

State Key Laboratory of Microbial Metabolism, School of Life Sciences and Biotechnology, Shanghai Jiao Tong University, Shanghai, 200240, PR China; Key Laboratory of Polar Ecosystem and Climate Change, Ministry of Education; School of Oceanography, Shanghai Jiao Tong University, Shanghai 200240, China

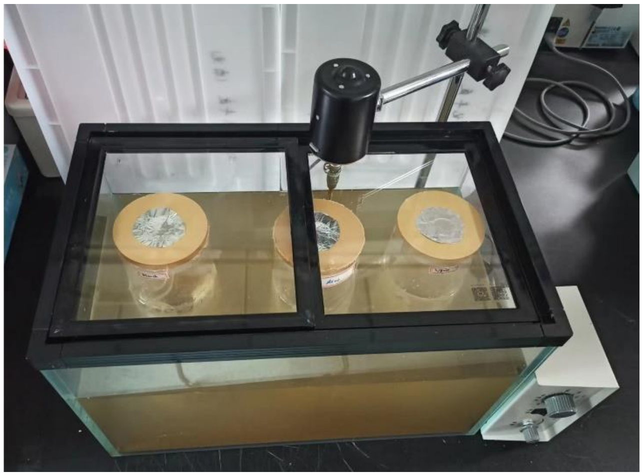

Fig. S1 A Photo of diffusion-based cultivation apparatus "Microbial Aquarium".

Table S1a. Relative abundance and bacterial taxonomic identification of initial-unamended and one-month enriched SCS sediment sample

Bacterial Phyla (SCS-sample)	Initial sample	One-month DICA enriched samples			One-month TCA enriched samples		
		Relative abundance (%)					
	Unamended	DICA-Lig	DICA-ASW	DICA-St	TCA-Lig	TCA-ASW	TCA-St
Candidatus Acetothermia	0.005	0.031	0.009	0.009	0.011	-	-
Acidobacteriota	0.044	0.059	0.194	0.008	0.014	0.002	0.001
Actinobacteriota	0.058	0.033	0.046	1.106	0.437	0.052	4.075
Candidatus Aerophobota	-	0.001	0.011	-	-	-	-
Armatimonadota	0.003	0.231	0.037	0.004	0.006	-	-
Bacillota	0.066	0.477	0.05	0.038	0.036	0.106	0.151
Bacteroidota	14.11	5.109	9.357	1.326	18.78	8.831	21.984
Bdellovibrionota	0.029	0.035	0.0254	0.102	0.137	-	-
Candidatus Caldatribacteriota	0.002	0.002	0.01	0.004	0.004	-	-
Chloroflexota	0.027	0.15	0.658	0.026	0.011	0.001	0.002
CK-2C2-2	0.001	-	0.003	0.001	-	-	-
Cyanobacteria	0.005	-	-	-	-	0.012	0.018
Deferribacterota	-	-	-	-	-	0.001	0.001
Desulfobacterota	0.325	57.67	0.384	0.18	0.189	-	-
Elusimicrobiota	0.013	0.031	0.033	0.01	0.005	-	-
Fusobacteriota	0.006	0.0158	0.004	0.002	0.003	0.001	-
Gemmatimonadota	0.002	0.028	0.005	0.005	0.044	-	-
Candidatus Hydrogenedentes	0.245	0.057	0.0039	0.08	0.184	-	-
Candidatus Latescibacterota	0.005	0.007	0.007	0.002	0.005	-	-
Myxococcota	0.021	0.041	0.021	0.018	0.005	-	-
NB1-j	0.002	0.001	0.003	0.001	0.003	-	-
Nitrospirota	0.011	0.02	0.007	0.006	0.008	-	-
Candidatus Patescibacteria	0.056	0.032	0.124	0.001	0.007	-	-
Planctomycetota	5.04	0.232	0.222	1.277	11.999	0.007	0.009
Pseudomonadota	79.6	35.31	88.57	83.762	67.98	80.562	60.679
Spirochaetota	0.001	0.1	0.0163	0.001	-	0.001	-
Sva0485	0.016	0.035	0.126	0.02	0.015	-	-
TA06	-	0.01	0.008	0.002	0.007	-	-
Unknown; unclassified	0.054	0.043	0.023	0.038	0.015	10.42	13.074
Verrucomicrobiota	0.22	0.071	0.024	0.018	0.072	-	0.001
Candidatus Zixibacteria	0.004	0.146	0.008	0.001	-	-	-

Table S1b. Relative abundance and bacterial taxonomic identification of initial-unamended and one-month enriched MT sediment sample

Bacterial Phyla (MT-sample)	Initial sample	One-month DICA enriched samples			One-month TCA enriched samples		
		Relative abundance (%)					
	Unamended	DICA-Lig	DICA-ASW	DICA-St	TCA-Lig	TCA-ASW	TCA-St
Acidobacteriota	0.001	0.003	0.002	0.004	0.007	0.001	0.062
Actinobacteriota	0.049	0.123	0.238	0.117	0.139	4.075	-
Armatimonadota	-	-	0.001	0.001	-	-	-
Bacillota	0.121	0.492	0.305	0.249	0.326	0.151	0.214
Bacteroidota	0.255	5.213	17.215	7.225	1.366	21.984	1.102
Balneolota	0.001	0.002	0.01	0.004	0.006	-	-
Chloroflexota	0.003	0.006	0.108	0.168	0.007	0.002	0.002
Cyanobacteria	0.012	0.024	0.025	-	0.023	0.018	0.04
Deinococcus-Thermus	-	0.001	-	-	0.001	-	-
Deferribacterota	-	0.019	-	0.001	0.002	0.001	-
Fusobacteriota	-	0.001	0.009	0.002	-	-	-
Gemmatimonadota	-	-	-	0.001	-	-	-
Planctomycetota	0.003	0.216	0.269	0.207	0.075	0.009	0.005
Pseudomonadota	72.185	62.041	52.199	56.474	61.939	60.679	48.02
Spirochaetota	-	0.331	0.002	0.002	0.004	-	-
Mycoplasmatota	-	0.003	0.004	0.001	0.003	-	-
Unknown; unclassified	27.345	31.524	29.592	35.49	36.094	13.074	50.546
Verrucomicrobiota	-	0.001	0.001	0.002	-	0.001	-

**Table S2 a. List of novel isolates obtained using the diffusion-based integrative cultivation approach (DICA) and Traditional cultivation approach (TCA)**						
S1 and S2 shows sediment samples from South China Sea and Mariana Trench respectively.						
Red color: new species candidate; Blue color: new genus candidate; Green color: new family candidate; Black color: previously uncultured						
**Strains code/ Genebank accession number**	**Closely related to uncultured bacteria/valid type species**	**16S rRNA similarity %**	**Taxonomy**	**Sample**	**Modified enrichment media**	**Incubation time on agar plates**
**Novel strains isolated using DICA**						
DICA-001/OQ055016	*Cephaloticoccus primus* strain CAG34 (NR_151907)	92	*Verrucomicrobiota*	S1	Lig	4 weeks
DICA-002/OQ055017	Uncultured bacterium clone 3C003229 (EU801852.1)	96.01	*Verrucomicrobiota*	S1	Lig	3 weeks
	Cephaloticoccus primus strain CAG34 (NR_151907)	94.65				
DICA -003/OQ055018	Uncultured bacterium clone: SC-144 (AB255109.1)	99.6	*Alphaproteobacteria*	S1	Lig	4 weeks
	Parvibaculum sedimenti strain HXT-9 (NR_180859)	97.27				
DICA-004/OQ055019	*Tepidicaulis marinus* strain MA2 (NR_135865)	94.61	*Alphaproteobacteria*	S1	Lig	3 weeks
DICA-005/OQ055020	Uncultured bacterium clone WC3_145 (GQ264048.1)	99.15	*Alphaproteobacteria*	S1	Lig	3 weeks
	Pseudolabrys taiwanensis strain CC-BB4 (NR_043515)	95.87				
DICA-006/OQ055021	Uncultured bacterium clone V1SC07b50 (HQ153947.1)	93.7	*Alphaproteobacteria*	S1	Lig	2 weeks
	Lutibaculum baratangense strain AMV1 (NR_116954)	91.27				
DICA-007/OQ055022	*Butyratibacter algicola* strain B15 (NR_157992)	89.83	*Alphaproteobacteria*	S1	Lig	3 weeks
DICA-008/OQ055023	Uncultured bacterium clone YD1600-64 (JX441418.1)	99.85	*Gammaproteobacteria*	S1	Lig	3 weeks
	Thiolapillus brandeum strain Hiromi (NR_148757)	93.29				
DICA-009/OQ055024	Uncultured bacterium clone F10_10.2_1 (FJ716861.1)	99.86	*Alphaproteobacteria*	S1	Lig	5 weeks
	Octadecabacter arcticus strain 238 (NR_102905)	96.43				
DICA-010/OQ055025	Uncultured bacterium clone BF_GA22-18 (KC238390.1)	99.79	*Alphaproteobacteria*	S1	Lig	5 weeks
	Varunaivibrio sulfuroxidans strain TC8 (NR_152005)	92.17				
DICA-011/OQ055026	Uncultured bacterium clone EzyYy191 (KX172283.1)	98.94	*Actinomycetota*	S1	Lig	~1.5 weeks
	Ilumatobacter fluminis strain YM22-133 (NR_041633)	97.27				

DICA-012/OQ055027	Uncultured bacterium clone WW3_66 (GQ264514)	97.79	*Alphaproteobacteria*	S1	Lig	~1.5 weeks
	Devosia insulae strain DS-56 (NR_044036)	97.35				
DICA-013/OQ055028	Uncultured alpha proteobacterium clone (LC171294.1)	96.91	*Alphaproteobacteria*	S1	Lig	1 week
	Salaquimonas pukyongi strain RR3-28 (NR_173673)	96.76				
DICA-014/OQ055029	*Aequorivita vladivostokensis* strain NBRC 16718 (NR_113866)	97.72	*Bacteroidota*	S1	Lig	7 weeks
DICA-015/OQ055030	Uncultured bacterium clone B169 (KJ817631.1)	96.38	*Alphaproteobacteria*	S1	Lig	5 weeks
	Litoreibacter meonggei strain MA1-1 (NR_109438)	96.09				
DICA-016/OQ055031	*Methylophaga thiooxydans* strain DMS010 (NR_115846)	97.08	*Gammaproteobacteria*	S1	Lig	1 week
DICA-017/OQ055032	Uncultured bacterium clone 16S22 (KX589511.1)	99.58	*Gammaproteobacteria*	S1	Lig	3 weeks
	Alloalcanivorax dieselolei strain B5 (NR_074734)	92.84				
DICA-018/OQ055033	*Arsenicitalea aurantiaca* strain 42-50 (NR_153745)	94.85	*Alphaproteobacteria*	S1	ASW	2 weeks
DICA-019/OQ055034	*Gracilimonas amylolytica* strain LA399 (NR_178892)	96.89	*Balneolota*	S2	Lig	3 weeks
DICA-020/OQ055035	Uncultured bacterium clone NCAAH 15N16 (KR537248.1)	98.65	*Actinomycetota*	S1	ASW	1 week
	Actinomarinicola tropica strain SCSIO 58843 (NR_171428)	90.41				
DICA-021/OQ055036	Uncultured bacterium clone JS5_521 (KR825135.1)	98.85	*Actinomycetota*	S1	Lig	1 week
	Actinomarinicola tropica strain SCSIO 58843 (NR_171428)	91.78				
DICA-022/OQ055037	Uncultured bacterium enrichment clone R1492-6 (JF449939.1)	100	*Alphaproteobacteria*	S1	Lig	2 weeks
	Tabrizicola fusiformis strain SY72 (NR_179727)	95.93				
DICA-023/OQ055038	Uncultured actinobacterium clone PET-130 (JF344254.1)	98.51	*Actinomycetota*	S1	Lig	7 weeks
	Ilumatobacter fluminis strain YM22-133 (NR_041633)	96.74				
DICA-024/OQ055039	Uncultured Rhodospirillales bacterium BPS_H570 (HQ857670.1)	99.93	*Alphaproteobacteria*	S1	Lig	2 weeks
	Oceanibaculum pacificum strain LMC2up-L3 (NR_116679)	89.96				
DICA-025/OQ055040	Uncultured bacterium clone: MPB2-25 (AB630701.1)	98.56	*Alphaproteobacteria*	S1	Lig	3 weeks
	Kaustia mangrovi strain R1DC25 (NR_173607)	87.63				
DICA-026/OQ055041	Uncultured alpha proteobacterium SR530ST15 (JN790964.1)	96.42	*Alphaproteobacteria*	S1	Lig	2 weeks
	Acuticoccus sediminis strain PTG4-2 (NR_178344)	95				
DICA-027/OQ055042	*Halophibacterium profundimaris* strain PC39 (NR_178412)	94.57	*Gammaproteobacteria*	S1	Lig	3 weeks
DICA-028/OQ055043	*Thalassobius aquimarinus* strain KMM 8518 (NR_179356)	97.17	*Alphaproteobacteria*	S1	Lig	1 week
DICA-029/OQ055044	*Agromyces mediolanus* strain VKM Ac-1388 (NR_118453)	97.74	*Actinomycetota*	S1	ASW	8 weeks

DICA-030/OQ055045	Uncultured alpha proteobacterium clone B02-03G (FJ542954.1)	98.55	*Alphaproteobacteria*	S1	ASW	2 weeks
	Pseudoxanthobacter soli strain CC4 (NR_044225)	94.85				
DICA-031/OQ055046	Uncultured bacterium clone P05CH1_59_61 (HF558572.1)	99.93	*Actinomycetota*	S1	Lig	1 week
	Demequina aestuarii strain JC2054 (NR_043465)	96.37				
DICA-032/OQ055047	Uncultured Bacteroidetes bacterium VS_CL-274 (FJ497526.1)	98.79	*Bacteroidota*	S2	Lig	1 week
	Lutibacter crassostreae strain TYO-8 (NR_136818)	96.88				
DICA-033/OQ055048	Uncultured bacterium clone S75-80-20 (KJ752783.1)	98.44	*Gammaproteobacteria*	S2	ASW	2 weeks
	Pseudohongiella spirulinae strain Ma-20 (NR_126265)	94.78				
DICA-034/OQ055049	Uncultured alpha proteobacterium MSB-4H3 (DQ811855.1)	95.84	*Alphaproteobacteria*	S2	ASW	2 weeks
	Fodinicurvata fenggangensis strain YIM D812 (NR_044596)	91.35				
DICA-035/OQ055050	*Tessaracoccus coleopterorum* strain HDW20 (NR_180655)	95.11	*Actinomycetota*	S2	St	2 weeks
DICA-036/OQ055051	Uncultured bacterium PropaneSIP6-6-11 (GU584713.1)	99.93	*Gammaproteobacteria*	S2	ASW	2 weeks
	Porticoccus hydrocarbonoclasticus strain MCTG13d (NR_118247)	95.48				
DICA-037/OQ055052	Aequorivita sinensis strain 8-1b (NR_109011)	97.66	*Bacteroidota*	S2	St	2 weeks
DICA-038/OQ200131	*Marileptolyngbya sina* 16S ribosomal RNA (NR_177705)	87.87	*Cyanobacteria*	S2	Lig	2 weeks
DICA-149/OQ062553	*Patulibacter ginsengiterrae* strain P4-5 (NR_108221)	98.65	*Thermoleophilia*	S1	Lig	2 weeks
DICA-040/OQ055054	Uncultured bacterium clone 33B FPP106A (LN875339.1)	98.23	*Bacteroidota*	S1	St	3 weeks
	Pontimicrobium aquaticum strain CAU 1491 (NR_175496)	96.59				
DICA-041/OQ055055	Uncultured bacterium clone S1B1S_11-003 (KY190873.1)	98.27	*Gammaproteobacteria*	S1	Lig	2 weeks
	Wenzhouxiangella marina strain Ma-11 (NR_136878)	91.54				
DICA-042/OQ055056	Uncultured bacterium clone AN7-6 (KJ590571.1)	99.37	*Betaproteobacteria*	S1	Lig	2 weeks
	Methylotenera mobilis strain JLW8 (NR_102842)	95.44				
DICA-043/OQ055057	*Muricauda aurea* strain BC31-1-A7 (NR_181667)	97.51	*Bacteroidota*	S1	Lig	3 weeks
DICA-044/OQ055058	Uncultured bacterium clone 16S22 (KX589511.1)	99.72	*Gammaproteobacteria*	S1	Lig	3 weeks
	Alloalcanivorax dieselolei strain B5 (NR_074734)	92.94				
DICA-045/OQ055059	*Methylophaga thiooxydans* strain DMS010 (NR_115846)	97.17	*Gammaproteobacteria*	S1	Lig	1 week
DICA-046/OQ055060	*Methylophaga thiooxydans* strain DMS010 (NR_115846)	97.11	*Gammaproteobacteria*	S1	Lig	1 week
DICA-047/OQ055061	*Methylophaga thiooxydans* strain DMS010 (NR_115846)	97.02	*Gammaproteobacteria*	S1	ASW	~1.5 weeks
DICA-048/OQ055062	*Methylophaga thiooxydans* strain DMS010 (NR_115846)	97.12	*Gammaproteobacteria*	S1	ASW	~1.5 weeks
DICA-049/OQ055063	*Methylophaga thiooxydans* strain DMS010 (NR_115846)	97.12	*Gammaproteobacteria*	S1	ASW	~1.5 weeks

DICA-050/OQ055064	*Methylophaga thiooxydans* strain DMS010 (NR_115846)	97.12	*Gammaproteobacteria*	S1	St	1 week
DICA-051/OQ055065	*Methylophaga thiooxydans* strain DMS010 (NR_115846)	97.13	*Gammaproteobacteria*	S1	St	1 week
DICA-052/OQ055066	Uncultured bacterium clone EzyYy191 (KX172283.1)	98.87	*Actinomycetota*	S1	St	~1.5 weeks
	Ilumatobacter fluminis strain YM22-133 (NR_041633)	97.3				
DICA-053/OQ055067	Uncultured bacterium clone EzyYy191 (KX172283.1)	98.88	*Actinomycetota*	S1	St	~1.5 weeks
	Ilumatobacter fluminis strain YM22-133 (NR_041633)	97.31				
DICA-054/OQ055068	Uncultured bacterium clone AKAU4191 (DQ125920.1)	98.95	*Alphaproteobacteria*	S1	Lig	3 weeks
	Pseudolabrys taiwanensis strain CC-BB4 (NR_043515)	95.68				
DICA-055/OQ055069	Uncultured bacterium clone AKAU4191 (DQ125920.1)	98.88	*Alphaproteobacteria*	S1	ASW	3 weeks
	Pseudolabrys taiwanensis strain CC-BB4 (NR_043515)	95.62				
DICA-056/OQ055070	Uncultured bacterium clone AKAU4191 (DQ125920.1)	98.95	*Alphaproteobacteria*	S1	ASW	3 weeks
	Pseudolabrys taiwanensis strain CC-BB4 (NR_043515)	95.68				
DICA-057/OQ055071	Uncultured bacterium clone NCAAH 15N16 (KR537248.1)	97.45	*Actinomycetota*	S1	Lig	1 week
	Actinomarinicola tropica strain SCSIO 58843 (NR_171428)	90.41				
DICA-058/OQ055072	Uncultured bacterium clone AN7-6 (KJ590571.1)	99.3	*Betaproteobacteria*	S1	Lig	
	Methylotenera mobilis strain JLW8 (NR_102842)	95.39				2 weeks
DICA-059/OQ055073	Uncultured alpha proteobacterium clone (LC171294.1)	96.91	*Alphaproteobacteria*	S1	ASW	1 week
	Salaquimonas pukyongi strain RR3-28 (NR_173673)	96.76				
DICA-060/OQ055074	Uncultured alpha proteobacterium clone (LC171294.1)	96.92	*Alphaproteobacteria*	S1	St	1 week
	Salaquimonas pukyongi strain RR3-28 (NR_173673)	97.77				
DICA-061/OQ055075	Uncultured alpha proteobacterium clone (LC171294.1)	96.93	*Alphaproteobacteria*	S1	ASW	1 week
	Salaquimonas pukyongi strain RR3-28 (NR_173673)	96.79				
DICA-062/OQ055076	Uncultured bacterium clone 16S22 (KX589511.1)	99.72	*Gammaproteobacteria*	S1	St	
	Alloalcanivorax dieselolei strain B5 (NR_074734)	92.96				3 weeks
DICA-063/OQ055077	Uncultured bacterium clone 16S22 (KX589511.1)	99.65	*Gammaproteobacteria*	S1	ASW	
	Alcanivorax indicus strain SW127 (NR_180069.1)	92.98				3 weeks
DICA-064/OQ055078	*Parvibaculum sedimenti* strain HXT-9 (NR_180859)	97.24	*Alphaproteobacteria*	S1	St	1 week
DICA-065/OQ055079	*Parvibaculum sedimenti* strain HXT-9 (NR_180859)	97.24	*Alphaproteobacteria*	S1	St	1 week
DICA-066/OQ055080	*Halophibacterium profundimaris* strain PC39 (NR_178412.1)	94.41	*Gammaproteobacteria*	S1	Lig	
DICA-067/OQ055081	Uncultured bacterium enrichment clone R1492-6 (JF449939.1)	99.93	*Alphaproteobacteria*	S1	St	2 weeks
	Tabrizicola fusiformis strain SY72 (NR_179727)	95.74				
DICA-068/OQ055082	Uncultured bacterium clone 3C003229 (EU801852.1)	96.35	*Verrucomicrobiota*	S1	Lig	3 weeks
	Cephaloticoccus primus strain CAG34 (NR_151907)	94.83				

DICA-069/OQ055083	Uncultured bacterium clone 3C003229 (EU801852.1)	96.2	*Verrucomicrobiota*	S1	Lig	3 weeks
	Cephaloticoccus primus strain CAG34 (NR_151907)	94.96				
DICA-70/OQ055084	*Gracilimonas amylolytica* strain LA399 (NR_178892)	96.89	*Balneolota*	S2	Lig	3 weeks
DICA-071/OQ055085	Uncultured bacterium clone Bms_MS122 (HQ697733.1)	97.04	*Alphaproteobacteria*	S2	Lig	2 weeks
	Novispirillum itersonii strain NBRC 15648 (NR_113793)	93.86				
DICA-072/OQ055086	*Minwuia thermotolerans* strain SY3-15 (NR_179861)	90.69	*Alphaproteobacteria*	S2	Lig	1 week
DICA-073/OQ055087	Uncultured bacterium clone Bms_MS122 (HQ697733.1)	97.1	*Alphaproteobacteria*	S2	Lig	2 weeks
	Novispirillum itersonii strain NBRC 15648 (NR_113793)	93.9				
DICA-074/OQ055088	*Lutibaculum pontilimi* strain GH1-34 (NR_179609)	98.62	*Alphaproteobacteria*	S2	ASW	2 weeks
DICA-075/OQ055089	Uncultured Bacteroidetes bacterium clone (FJ497526.1)	98.57	*Bacteroidota*	S2	ASW	1 week
	Lutibacter crassostreae strain TYO-8 (NR_136818)	96.65				
DICA-076/OQ055090	*Demequina salsinemoris* strain NBRC 105323 (NR_113372)	98.08	*Actinomycetota*	S2	Lig	1 week
DICA-077/OQ055091	Uncultured bacterium clone 3 (HQ893767.1)	99.93	*Alphaproteobacteria*	S2	Lig	1 week
	Stappia stellulata strain NBRC 15764 (NR_113809)	98.54				
DICA-078/OQ055092	Uncultured bacterium clone Bms_MS122 (HQ697733.1)	96.87	*Alphaproteobacteria*	S2	ASW	2 weeks
	Novispirillum itersonii strain NBRC 15648 (NR_113793)	92.75				
DICA-079/OQ055093	Uncultured bacterium clone Bms_MS122 (HQ697733.1)	96.95	*Alphaproteobacteria*	S2	Lig	2 weeks
	Novispirillum itersonii strain NBRC 15648 (NR_113793)	93.83				
DICA-080/OQ055094	*Minwuia thermotolerans* strain SY3-15 (NR_179861)	90.64	*Alphaproteobacteria*	S2	Lig	1 week
DICA-081/OQ055095	*Minwuia thermotolerans* strain SY3-15 (NR_179861)	90.65	*Alphaproteobacteria*	S2	Asw	1 week
DICA-082/OQ055096	*Minwuia thermotolerans* strain SY3-15 (NR_179861)	90.94	*Alphaproteobacteria*	S2	Asw	1 week
DICA-083/OQ055097	Uncultured bacterium clone NC55 (KJ590695.1)	99.85	*Alphaproteobacteria*	S1	ASW	~2.5 weeks
	Magnetospira thiophila strain MMS-1 (NR_116475)	91.57				
DICA-084/OQ055098	*Demequina salsinemoris* strain NBRC 105323 (NR_113372)	98.28	*Actinomycetota*	S2	Lig	1 week
DICA-085/OQ055099	*Minwuia thermotolerans* strain SY3-15 (NR_179861)	90.65	*Alphaproteobacteria*	S2	St	1 week
DICA-086/OQ055100	*Tessaracoccus nasisuum* strain 1a6R-CH11an (NR_179986)	97.49	*Actinomycetota*	S2	Lig	
DICA-087/OQ055101	Uncultured bacterium clone NC55 (KJ590695.1)	99.86	*Alphaproteobacteria*	S2	Lig	~2.5 weeks
	Magnetospira thiophila strain MMS-1 (NR_116475)	91.57				
DICA-088/OQ055102	Uncultured bacterium clone AN7-6 (KJ590571.1)	99.3	*Betaproteobacteria*	S2	St	2 weeks
	Methylotenera mobilis strain JLW8 (NR_102842)	95.37				
DICA-089/OQ055103	*Demequina salsinemoris* strain NBRC 105323 (NR_113372)	98.09	*Actinomycetota*	S2	ASW	1 week
DICA-090/OQ055104	Uncultured bacterium clone NC55 (KJ590695.1)	99.71	*Alphaproteobacteria*	S2	St	2 weeks
	Magnetospira thiophila strain MMS-1 (NR_116475)	91.5				

DICA-091/OQ055105	*Kordiimonas aestuarii* strain 101-1 (NR_109376)	98.48	*Alphaproteobacteria*	S2	ASW	1 week
DICA-092/OQ055106	*Minwuia thermotolerans* strain SY3-15 (NR_179861)	90.67	*Alphaproteobacteria*	S2	ASW	1 week
DICA-093/OQ055107	Uncultured bacterium clone NC55 (KJ590695.1)	99.71	*Alphaproteobacteria*	S2	ASW	2 weeks
	Magnetospira thiophila strain MMS-1 (NR_116475)	91.57				
DICA-094/OQ055108	Uncultured bacterium clone AN7-6 (KJ590571.1)	99.36	*Betaproteobacteria*	S2	Lig	2 weeks
	Methylotenera mobilis strain JLW8 (NR_102842)	95.38				
DICA-095/OQ055109	Uncultured bacterium clone 3 (HQ893767.1)	99.85	*Alphaproteobacteria*	S2	Lig	1 week
	Stappia stellulata strain NBRC 15764 (NR_113809)	98.48				
DICA-096/OQ055110	*Demequina salsinemoris* strain NBRC 105323 (NR_113372)	98.26	*Actinomycetota*	S2	St	1 week
DICA-097/OQ055111	*Mesorhizobium olivaresii* strain CPS13 (NR_149815)	96.93	*Alphaproteobacteria*	S2	ASW	2 week
DICA-098/OQ055112	*Demequina salsinemoris* strain NBRC 105323 (NR_113372)	98.15	*Actinomycetota*	S2	ASW	1 week
DICA -099/OQ055113	Uncultured bacterium clone 3 (HQ893767.1)	99.85	*Alphaproteobacteria*	S2	St	1 week
	Stappia stellulata strain NBRC 15764 (NR_113809)	98.46				
DICA-100/OQ055114	*Demequina salsinemoris* strain NBRC 105323 (NR_113372)	98.08	*Actinomycetota*	S2	ASW	1 week
DICA-101/OQ055116	*Demequina salsinemoris* strain NBRC 105323 (NR_113372)	98.15	*Actinomycetota*	S2	St	1 week
DICA-102/OQ055117	Uncultured bacterium clone 3 (HQ893767.1)	99.64	*Alphaproteobacteria*	S2	St	1 week
	Stappia stellulata strain NBRC 15764 (NR_113809)	98.27				
DICA-103/OQ055118	*Hoeflea halophila* strain JG120-1 (NR_108835)	98.48	*Alphaproteobacteria*	S2	ASW	1 week
DICA-115/OQ062519	*Patulibacter ginsengiterrae* strain P4-5 (NR_108221)	98.52	*Actinomycetota*	S2	Lig	2 weeks
DICA-105/OQ055120	Uncultured bacterium clone S26-15 (EU287315.1)	98.06	*Alphaproteobacteria*	S2	Lig	2 weeks
	Pseudemcibacter aquimaris strain Y4 (NR_181789)	96.92				
DICA-106/OQ055121	*Muricauda aurea* strain BC31-1-A7 (NR_181667)	97.4	*Bacteroidota*	S2	St	3 weeks
DICA-107/OQ055122	*Sphingomonas zeicaulis* strain 541 (NR_152012)	97.38	*Alphaproteobacteria*	S2	Lig	1 week
DI CA-108/OQ055123	Uncultured bacterium clone AN7-6 (KJ590571.1)	99.35	*Betaproteobacteria*	S2	St	2 weeks
	Methylotenera mobilis strain JLW8 (NR_102842)	95.35				
DICA-109/OQ055124	*Fulvivirga lutimaris* strain TM-6 (NR_149792)	98.2	*Bacteroidota*	S2	Lig	8 weeks
DICA-110/OQ055125	*Gaetbulibacter aquiaggeris* strain KEM-8 (NR_148804)	97.51	*Bacteroidota*	S2	Lig	1 week
DICA-111/OQ055126	*Rhodobium orientis* strain MB312 (NR_029128)	92.68	*Alphaproteobacteria*	S2	Lig	3 weeks
DICA-112/OQ055127	*Actinomycetospora chlora* strain TT07I-57 (NR_112968)	98.29	*Actinomycetota*	S2	St	1 week
DICA-113/OQ055128	*Rhodobium orientis* strain MB312 (NR_029128)	92.6	*Alphaproteobacteria*	S2	Lig	3 weeks
DICA-114/OQ055129	*Rhodobium orientis* strain MB312 (NR_029128)	92.6	*Alphaproteobacteria*	S2	ASW	3 weeks
DICA-137/OQ062541	*Hoeflea halophila* strain JG120-1 (NR_108835)	98.6	*Alphaproteobacteria*	S2	St	1 week

**Novel strains isolated using TCA**						
TCA-01/OQ055130	*Shewanella sediminis* strain HAW-EB3 (NR_074819)	97.97	*Gammaproteobacteria*	S1	Lig	1 week
TCA-02/OQ055131	*Demequina salsinemoris* strain NBRC 105323 (NR_113372)	98.09	*Actinomycetota*	S2	ASW	1 week
TCA-03/OQ055132	*Devosia naphthalenivorans* strain CM5-1 (NR_171486)	97.95	*Alphaproteobacteria*	S1	ASW	1 week
TCA-04/OQ055133	*Devosia yakushimensis* strain NBRC 103855 (NR_114252)	97.8	*Alphaproteobacteria*	S1	St	1 week
TCA-05/OQ055134	*Breoghania corrubedonensis* strain UBF-P1 (NR_104495)	95.46	*Alphaproteobacteria*	S2	Lig	1 week
TCA-07/OQ055136	*Anderseniella baltica* strain BA141 (NR_042626)	97.88	*Alphaproteobacteria*	S1	Lig	2 week
TCA-08/OQ055137	*Oricola cellulosilytica* strain CC-AMH-0 (NR_148297)	97.6	*Alphaproteobacteria*	S1	Lig	2 weeks
TCA-09/OQ055138	*Halomonas ilicicola* strain SP8 (NR_044436)	97.42	*Gammaproteobacteria*	S1	ASW	1 week
TCA-10/OQ055139	*Halomonas ilicicola* strain SP8 (NR_044436)	97.7	*Gammaproteobacteria*	S1	Lig	1 week
TCA-14/OQ055143	Uncultured gamma proteobacterium clone B97 (JQ753147.1)	99.79	*Gammaproteobacteria*	S2	Lig	2 weeks
	Methylophaga nitratireducenticrescens strain JAM1(NR_074321)	98.53				
TCA-15/OQ055144	Uncultured bacterium clone 3 (HQ893767.1)	100	*Alphaproteobacteria*	S2	Lig	1 week
	Stappia stellulata strain NBRC 15764 (NR_113809)	98.6				
TCA -16/OQ055145	Uncultured bacterium clone P05CH1_59_61 (HF558572.1)	99.93	*Actinomycetota*	S2	ASW	~1.5 weeks
	Demequina lutea strain SV45 (NR_044222)	98.43				
TCA-17/OQ055146	Uncultured bacterium clone 3 (HQ893767.1)	100	*Alphaproteobacteria*	S2	Lig	1 week
	Stappia stellulata strain NBRC 15764 (NR_113809)	98.6				
TCA-18/OQ055147	*Demequina salsinemoris* strain NBRC 105323 (NR_113372)	98.1	*Actinomycetota*	S2	St	1 week
TCA -19/OQ055148	Uncultured bacterium clone 3 (HQ893767.1)	100	*Alphaproteobacteria*	S2	St	1 week
	Stappia stellulata strain NBRC 15764 (NR_113809)	98.6				
TCA-20/OQ055149	*Demequina salsinemoris* strain NBRC 105323 (NR_113372)	97.79	*Actinomycetota*	S2	St	1 week
TCA -21/OQ055150	Uncultured bacterium clone 3 (HQ893767.1)	99.93	*Alphaproteobacteria*	S2	ASW	1 week
	Stappia stellulata strain NBRC 15764 (NR_113809)	98.53				
TCA-23/OQ055152	*Pseudomonas borbori* strain R-20821(NR_042450)	98.41	*Gammaproteobacteria*	S2	ASW	1 week
TCA-24/OQ055153	*Paraglaciecola arctica* strain BSs20135 (NR_116288)	98.54	*Gammaproteobacteria*	S2	St	1 week
TCA-25/OQ055154	Uncultured bacterium clone AKAU4191 (DQ125920.1)	98.95	*Alphaproteobacteria*	S2	Lig	3 weeks
	Pseudolabrys taiwanensis strain CC-BB4 (NR_043515)	95.56				

Table S2b. List of known isolates obtained using the diffusion-based integrative cultivation approach (DICA) and Traditional cultivation approach (TCA)				
S1 and S2 shows sediment sample from South China Sea and Mariana Trench respectively.				
**Strains code/ Genebank accession number**	**Closest hit strains**	**16S rRNA similarity %**	**Sample**	**Modified enrichment media**
**Known strains isolated using DICA**				
DICA-116/OQ062520	*Maribacter luteus* strain RZ05	99.86	S2	ASW
DICA-117/OQ062521	*Parvibaculum lavamentivorans* strain DS-1	99.12	S2	Lig
DICA-118/OQ062522	*Pusillimonas maritima* strain 17-4A	100	S2	ASW
DICA-119/OQ062523	*Parvibaculum hydrocarboniclasticum* strain EPR92	99.85	S2	Lig
DICA-120/OQ062524	*Methylophaga nitratireducenticrescens* strain JAM1	98.87	S2	ASW
DICA-121/OQ062525	*Methylophaga nitratireducenticrescens* strain JAM1	98.59	S2	St
DICA-122/OQ062526	*Celeribacter baekdonensis* strain L-6	99.92	S2	ASW
DICA-123/OQ062527	*Enterococcus faecalis strain ATCC 19433*	99.93	S2	ASW
DICA-124/OQ062528	*Roseomonas mucosa* strain MDA5527	100	S2	ASW
DICA-125/OQ062529	*Tistrella bauzanensis* strain BZ78	98.92	S1	ASW
DICA-126/OQ062530	*Martelella mediterranea* strain MACL11	98.97	S2	Lig
DICA-127/OQ062531	*Pantoea dispersa strain DSM 30073*	99.37	S2	ASW
DICA-128/OQ062532	*Celeribacter baekdonensis* strain L-6	99.7	S2	Lig
DICA-129/OQ062533	*Stutzerimonas kunmingensis* strain HL22-2	99.65	S2	St
DICA-130/OQ062534	*Brucella anthropi* ATCC 49188	99.85	S2	Lig
DICA-131/OQ062535	*Thalassospira permensis* NBRC 106175 strain SMB34	99.78	S2	ASW
DICA-132/OQ062536	*Bacillus paranthracis* strain MCCC 1A00395	100	S2	Lig
DICA-133/OQ062537	*Micrococcus yunnanensis* strain YIM 65004	99.79	S2	ASW
DICA-134/OQ062538	*Lutibaculum pontilimi* strain GH1-34	98.9	S2	ASW
DICA-104/OQ055119	*Aequorivita iocasae* strain KX20305	99.93	S2	Lig
DICA-135/OQ062539	*Alloalcanivorax dieselolei* strain B5	99.65	S2	St
DICA-136/OQ062540	*Roseovarius halotolerans strain HJ50*	99.47	S2	St
DICA-138/OQ062542	*Phaeocystidibacter marisrubri* strain G18	99.65	S2	Lig
DICA-139/OQ062543	*Pseudonocardia carboxydivorans* strain Y8	99.58	S2	Lig

DICA-140/OQ062544	*Phaeocystidibacter marisrubri* strain G18	99.79	S2	Lig
DICA-141/OQ062545	*Roseovarius halotolerans strain HJ50*	99.55	S2	Lig
DICA-142/OQ062546	*Muricauda beolgyonensis* strain BB-My12	100	S2	St
DICA-143/OQ062547	*Muricauda beolgyonensis* strain BB-My12	100	S2	Lig
DICA-144/OQ062548	*Bacillus mobilis* strain MCCC 1A05942	99.72	S2	St
DICA-145/OQ062549	*Antarcticimicrobium luteum* strain 318-1	100	S2	St
DICA-146/OQ062550	*Pusillimonas maritima* strain 17-4A	99.5	S2	Lig
DICA-147 /OQ062551	*Amorphus suaedae* strain YC6899	99.14	S2	St
DICA-148/OQ062552	*Algoriphagus winogradskyi* strain LMG 21969	99.07	S2	ASW
DICA-039/OQ055053	*Patulibacter ginsengiterrae* strain P4-5 16S	98.66	S1	ASW
DICA-150/OQ062554	*Nocardioides exalbidus* strain RC825	98.76	S2	ASW
DICA-151/OQ062555	*Halomonas titanicae* BH1	99.72	S1	St
DICA-152/OQ062556	*Alteromonas stellipolaris* strain R10SW13	99.64	S1	ASW
DICA-153/OQ062557	*Paenibacillus polymyxa* strain DSM 36	99.48	S1	ASW
DICA-154/OQ062558	*Photobacterium frigidiphilum* strain SL13	99.16	S1	Lig
DICA-155 /OQ062559	*Planococcus citreus* strain NBRC 15849	100	S1	ASW
DICA-156/OQ062560	*Marinobacter profundi* strain PWS21	99.93	S1	St
DICA-157/OQ062561	*Sulfitobacter faviae* strain S5-53	99.85	S1	ASW
DICA-158/OQ062562	*Halomonas meridiana* strain DSM 5425	100	S1	ASW
DICA-159/OQ062563	*Parvibaculum lavamentivorans* strain DS-1	99.12	S2	St
DICA-160/OQ062564	*Microbacterium schleiferi* strain DSM 20489	99.93	S2	ASW
DICA-161/OQ062565	*Cyclobacterium amurskyense* strain KMM 6143	99.26	S2	ASW
DICA-162/OQ062566	*Parvibaculum indicum* strain P31	98.67	S2	St
DICA-163/OQ062567	*Martelella mediterranea* strain MACL11	99.14	S2	ASW
DICA-164/OQ062568	*Kocuria assamensis* strain S9-65	99.57	S1	ASW
DICA-165/OQ062569	*Salinibacterium amurskyense* strain KMM 3673	99.57	S1	ASW
DICA-166/OQ062570	*Brachybacterium paraconglomeratum* strain LMG 19861	99.79	S1	ASW
DICA-167/OQ062571	*Dietzia timorensis* strain ID05-A0528	100	S1	ASW
DICA-168/OQ062572	*Agromyces indicus* strain NIO-1018	99.3	S1	St
DICA-169/OQ062573	*Bauldia litoralis* strain 524-16	99.79	S1	St
DICA-170/OQ062574	*Alloalcanivorax xenomutans* strain JC109	99.86	S1	St
DICA-171/OQ062575	*Thalassospira profundimaris* WP0211	99.49	S1	St

DICA-172/OQ062576	*Parasphingopyxis lamellibrachiae* strain JAMH 0132	100	S1	St
DICA-173/OQ062577	*Microbacterium aoyamense* strain KV-492	98.79	S1	St
DICA-174/OQ062578	*Maricaulis salignorans* strain MCS 18	99.49	S1	St
DICA-175/OQ062579	*Pseudoglutamicibacter cumminsii* strain DMMZ 445	99.33	S1	St
DICA-176/OQ062580	*Moraxella osloensis* strain A1920	98.94	S1	St
DICA-177/OQ062581	*Filomicrobium insigne* strain SLG5B-19	99.78	S1	St
DICA-178/OQ062582	*Parvibaculum lavamentivorans* strain DS-1	99.06	S1	St
DICA-179/OQ062583	*Brevirhabdus pacifica* strain DY6-4	99.04	S1	St
DICA-180/OQ062584	*Staphylococcus saprophyticus subsp. saprophyticus* ATCC 15305	99.93	S1	St
DICA-181/OQ062585	*Marinobacter adhaerens* HP15	99.86	S1	St
DICA-182/OQ062586	*Amorphus suaedae* strain YC6899	99.93	S1	St
DICA-183/OQ062587	*Micrococcus endophyticus* strain YIM 56238	98.79	S2	Lig
DICA-184/OQ062588	*Corynebacterium doosanense* strain CAU 212	98.94	S2	Lig
DICA-185/OQ062589	*Sphingobium xenophagum* strain C1	98.84	S2	Lig
DICA-186/OQ062590	*Georgenia muralis* strain NBRC 103560	99.72	S2	Lig
DICA-187/OQ062591	*Citrimicrobium luteum* strain CBA4602	99.28	S2	St
DICA-188/OQ062592	*Pseudorhizobium marinum* strain MGL06	99.78	S2	St
DICA-189/OQ062593	*Qipengyuania aerophila* strain GH25	99.77	S2	St
DICA-190/OQ062594	*Alloalcanivorax gelatiniphagus* strain MEBiC08158	99.79	S2	St
DICA-191/OQ062595	*Bacillus altitudinis 41KF2b*	100	S2	St
DICA-192/OQ062596	*Streptomyces nigra* strain 452	99.72	S2	St
DICA-193/OQ062597	*Parvibaculum indicum* strain P31	99.78	S2	St
DICA-194/OQ062598	*Nitratireductor arenosus* strain CAU 1489	99.42	S2	ASW
DICA-195/OQ062599	*Parvibaculum indicum* strain P31	99.78	S2	St
DICA-196/OQ062600	*Microbacterium algeriense* strain G1	99.58	S1	St

**Known strains isolated using TCA**				
TCA-026/OQ071638	*Alloalcanivorax venustensis* strain ISO4	99.3	S2	ASW
TCA-027/OQ071639	*Amorphus suaedae* strain YC6899	99.27	S2	ASW
TCA-028/OQ071640	*Parvibaculum lavamentivorans* strain DS-1	98.97	S2	ASW
TCA-029/OQ071641	*Parvibaculum lavamentivorans* strain DS-2	98.90	S2	Lig
TCA-030 /OQ071642	*Methylophaga nitratireducenticrescens* strain JAM2	98.67	S2	ASW
TCA-031/OQ071643	*Martelella mediterranea* strain MACL11	99.12	S2	ASW
TCA-032/OQ071644	*Alcanivorax gelatiniphagus* strain MEBiC08158	99.79	S2	ASW
TCA-033/OQ071645	*Pusillimonas maritima* strain 17-4A	100	S2	Lig
TCA-034/OQ071646	*Parvibaculum hydrocarboniclasticum* strain EPR92	99.6	S2	Lig
TCA-035/OQ071647	*Microbacterium schleiferi* strain DSM 20489	99.93	S2	ASW
TCA-036/OQ071648	*Methyloceanibacter caenitepidi* strain Gela4	99.27	S2	Lig
TCA-037/OQ071649	*Parvibaculum lavamentivorans* strain DS-1	98.9	S2	Lig
TCA-038/OQ071650	*Methylophaga nitratireducenticrescens* strain JAM1	98.94	S2	ASW
TCA-039/OQ071651	*Alloalcanivorax venustensis* strain ISO4	100	S2	Lig
TCA-040/OQ071652	*Idiomarina fontislapidosi* strain F23	99.29	S2	ASW
TCA-041/OQ071653	*Stutzerimonas kunmingensis* strain B13	99.58	S2	Lig
TCA-042/OQ071654	*Kordiimonas aestuarii* strain 101-1	98.69	S2	St
TCA-043/OQ071655	*Martelella mediterranea* strain MACL11	99.14	S2	Lig
TCA-044/OQ071656	*Methylophaga nitratireducenticrescens* strain JAM1	98.92	S2	ASW
TCA-045/OQ071657	*Pseudomonas songnenensis* strain NEAU-ST5-5	99.35	S2	Lig
TCA-046/OQ071658	*Parvibaculum indicum* strain P31	98.68	S2	Lig
TCA-047/OQ071659	*Alloalcanivorax marinus* strain R8-12	99.86	S2	Lig
TCA-048/OQ071660	*Parvibaculum lavamentivorans* strain DS-1	98.84	S2	Lig
TCA-049/OQ071661	*Alloalcanivorax gelatiniphagus* strain MEBiC08158	99.86	S2	St
TCA-050/OQ071662	*Alloalcanivorax dieselolei* strain B5	99.79	S2	St
TCA-051/OQ071663	*Marinobacter shengliensis* strain SL013A34A2	99.71	S2	St
TCA-052/OQ071664	*Pseudoalteromonas lipolytica* strain LMEB 39	99.71	S2	St
TCA-053/OQ071665	*Halomonas titanicae* strain BH1	99.65	S2	St
TCA-054 /OQ071666	*Microbacterium algeriense* strain G1	99.78	S2	Lig

TCA-055/OQ071667	*Aurantimonas coralicida* DSM 14790	100	S2	St
TCA-056/OQ071668	*Nitratireductor arenosus* strain CAU 1489	99.56	S2	St
TCA-057/OQ071669	*Halalkalibacterium halodurans* strain DSM 497	100	S2	St
TCA-058 /OQ071670	*Algoriphagus winogradskyi* strain LMG 21969	98.73	S2	St
TCA-059/OQ071671	*Roseovarius halotolerans* strain HJ50	99.34	S2	Lig
TCA-060/OQ071672	*Staphylococcus hominis subsp.novobiosepticus* strain GTC 1228	99.56	S2	Lig
TCA-061/OQ071673	*Alloalcanivorax marinus* strain R8-12	99.79	S2	Lig
TCA-062/OQ071674	*Roseovarius halotolerans strain HJ50*	99.55	S2	Lig
TCA-063/OQ071675	*Bauldia litoralis* strain 524-16	99.85	S2	Lig
TCA-064/OQ071676	*Pseudonocardia carboxydivorans* strain Y8	99.72	S2	St
TCA-065/OQ071677	*Streptomyces nigra* strain 452	99.72	S1	ASW
TCA-066/OQ071678	*Micrococcus yunnanensis* strain YIM 65004	99.71	S1	St
TCA-067/OQ071679	*Metabacillus idriensis* strain SMC 4352-2	100	S1	St
TCA-068/OQ071680	*Pseudoalteromonas agarivorans* strain DSM 14585	100	S1	St
TCA-069/OQ071681	*Marinobacter nauticus* strain ATCC 49840	100	S1	ASW
TCA-070/OQ071682	*Vibrio splendidus* strain AlyHP32	99.3	S1	Lig
TCA-071/OQ071683	*Vibrio gigantis* strain LGP 13	99.65	S1	Lig
TCA-072/OQ071684	*Halomonas litopenaei* strain SYSU ZJ2214	99.93	S1	Lig
TCA-073/OQ071685	*Sulfitobacter faviae* strain S5-53	99.85	S1	Lig
TCA-074/OQ071686	*Photobacterium indicum* strain MBIC3157	99.42	S1	Lig
TCA-075/OQ071687	*Marinobacter lipolyticus* strain SM-19	98.88	S1	ASW
TCA-076/OQ071688	*Pseudoalteromonas lipolytica* strain LMEB 39	99.93	S1	Lig
TCA-077/OQ071689	*Vibrio splendidus* strain AlyHP32	99.23	S1	Lig
TCA-078/OQ071690	*Halomonas piezotolerans* strain NBT06E8	99.93	S1	ASW
TCA-079/OQ071691	*Methylophaga thalassica* strain NBRC 102424	99.93	S1	ASW
TCA-080/OQ071692	*Salinicola salarius* strain M27	99.44	S1	Lig
TCA-081/OQ071693	*Halopseudomonas aestusnigri* strain VGXO14	99.86	S1	Lig
TCA-082/OQ071694	*Yangia mangrovi* strain SAOS 153D	99.85	S1	Lig
TCA-083/OQ071695	*Pseudoalteromonas arabiensis* strain k53	99.93	S1	Lig
TCA-084/OQ071696	*Pusillimonas maritima* strain 17-4A	100	S2	Lig
TCA-085/OQ071697	*Pseudoalteromonas tetraodonis* GFC strain KMM 458	99.8	S1	ASW
TCA-086/OQ071711	*Pseudoalteromonas arabiensis* strain k53	99.93	S2	ASW

TCA-087/OQ071712	*Vibrio gallaecicus* strain CECT 7244	99.93	S2	Lig
TCA-088/OQ071713	*Jeotgalibacillus terrae* strain JSM 081008	99.02	S1	ASW
TCA-089/OQ071714	*Staphylococcus pasteuri* strain ATCC 51129	100	S2	ASW
TCA-090 /OQ071715	*Exiguobacterium arabatum* strain RFL1109	99.93	S1	ASW
TCA-091/OQ071716	*Halomonas piezotolerans* strain NBT06E8	99.93	S1	ASW
TCA-092/OQ071717	*Halomonas litopenaei* strain SYSU ZJ2214(T)	99.93	S2	ASW
TCA-093/OQ071718	*Exiguobacterium arabatum* strain RFL1109	99.93	S2	ASW
TCA-094/OQ071719	*Parvibaculum lavamentivorans* strain DS-1	99.12	S1	ASW
TCA-095/OQ071720	*Yangia mangrovi* strain SAOS 153D	99.85	S2	ASW
TCA-096/OQ071721	*Amorphus suaedae* strain YC6899	99.13	S2	ASW
TCA-097/OQ071722	*Parvibaculum indicum* strain P31	98.74	S1	ASW
TCA-098/OQ071723	*Bauldia litoralis* strain 524-16	99.71	S2	Lig
TCA-099/OQ071724	*Methylophaga nitratireducenticrescens* strain JAM1	98.59	S1	Lig
TCA-100/OQ071725	*Pseudoalteromonas shioyasakiensis* strain SE3	98.66	S2	Lig
TCA-101/OQ071726	*Pseudoalteromonas shioyasakiensis* strain SE3	98.88	S1	Lig
TCA-102/OQ071727	*Pseudomonas songnenensis* strain NEAU-ST5-5	99.44	S2	ASW
TCA-103/OQ071728	*Halomonas meridiana* strain DSM 5425	100	S1	Lig
TCA-104/OQ071729	*Alcanivorax jadensis* strain T9	99.65	S1	Lig
TCA-105/OQ071730	*Alcanivorax jadensis* strain T9	99.65	S1	Lig
TCA-106/OQ071731	*Martelella mediterranea* strain MACL11	99.11	S1	ASW
TCA-107/OQ071732	*Photobacterium indicum* strain MBIC3157	99.49	S1	ASW
TCA-108/OQ071733	*Vibrio splendidus* strain AlyHP32	99.36	S1	Lig
TCA-109 /OQ071734	*Vibrio gallaecicus* strain CECT 7244	98.93	S1	Lig
TCA-110/OQ071735	*Stutzerimonas stutzeri* strain CCUG 11256	99.07	S1	ASW
TCA-111/OQ071736	*Alloalcanivorax gelatiniphagus* strain MEBiC08158	99.86	S1	ASW
TCA-112/OQ071737	*Bauldia litoralis* strain 524-16	99.81	S1	Lig
TCA-113/OQ071738	*Alloalcanivorax venustensis* strain ISO4	99.93	S2	ASW
TCA-114/OQ071739	*Thalassospira xiamenensis* strain M-5	99.49	S1	St
TCA-115/OQ071740	*Microbacterium profundi* strain Shh49	99.43	S2	Lig
TCA-116/OQ071741	*Idiomarina fontislapidosi* strain F23	99.15	S1	ASW
TCA-117/OQ071742	*Idiomarina fontislapidosi* strain F23	99.79	S1	ASW
TCA-118/OQ071743	*Alloalcanivorax venustensis* strain ISO4	99.86	S2	Lig

TCA-119/OQ071744	*Stutzerimonas kunmingensis* strain HL22-2	99.22	S2	ASW
TCA-120/OQ071745	*Qipengyuania aerophila* strain GH25	100	S1	Lig
TCA-121/OQ071746	*Stutzerimonas xanthomarina* strain KMM 1447	98.87	S1	ASW
TCA-122/OQ071747	*Methyloceanibacter caenitepidi* strain Gela4	99.27	S1	ASW
TCA-123/OQ071748	*Staphylococcus epidermidis* strain NBRC 100911	99.93	S1	ASW
TCA-124/OQ071749	*Parvibaculum indicum* strain P31	98.69	S1	Lig
TCA-06/OQ055135	*Oricola indica* strain JL-62	99.24	S1	Lig
TCA-125/OQ071750	*Marinobacter nauticus* ATCC 49840	100	S1	St
TCA-126/OQ071751	*Staphylococcus nepalensis* strain CW1	99.37	S1	St
TCA-127/OQ071752	*Sulfitobacter pontiacus* strain ChLG-10	99.7	S1	St
TCA-128/OQ071753	*Pseudomonas kuykendallii* strain H2	99.79	S1	St
TCA-129/OQ071754	*Brachybacterium paraconglomeratum* strain LMG 19861	99.41	S1	St
TCA-130/OQ071755	*Staphylococcus epidermidis* strain NBRC 100911	99.72	S1	St
TCA-131/OQ071756	*Alloalcanivorax dieselolei* strain B5	99.79	S1	St
TCA-132/OQ071757	*Qipengyuania aerophila* strain GH25	99.93	S1	St
TCA-133/OQ071758	*Stutzerimonas kunmingensis* strain HL22-2	99.37	S1	St
TCA-134/OQ071759	*Pusillimonas maritima* strain 17-4A	98.88	S1	St
TCA-135/OQ071760	*Ornithinimicrobium panacihumi* strain DCY118	98.94	S1	St
TCA-136/OQ071761	*Micrococcus yunnanensis* strain YIM 65004	99.57	S1	St
TCA-137/OQ071762	*Kordiimonas aestuarii* strain 101-1	98.89	S2	St
TCA-138/OQ071763	*Thalassospira xiamenensis* strain M-5	99.49	S1	St
TCA-139/OQ071764	*Pusillimonas maritima* strain 17-4A	99.02	S1	St
TCA-140/OQ071765	*Nocardioides ganghwensis* strain JC2055	99.85	S1	St
TCA-141/OQ071766	*Parasphingorhabdus flavimaris* strain SW-151	99.93	S1	St
TCA-142/OQ071767	Geodermatophilus nigrescens strain DSM 45408	99.71	S1	St
TCA-143/OQ071768	*Oricola indica* strain JL-62	98.83	S1	St
TCA-144/OQ071769	*Nitratireductor arenosus* strain CAU 1489	99.56	S1	St
TCA-145/OQ071770	*Jannaschia helgolandensis* strain Hel 10	100	S1	St
TCA-146/OQ071771	*Thalassolituus oleivorans* strain MIL-1	99.72	S1	St
TCA-147/OQ071772	*Lutibaculum pontilimi* strain GH1-34	98.77	S1	St
TCA-148/OQ071773	*Pseudomonas marincola* strain AB251f	98.79	S1	St
TCA-149/OQ071774	*Kocuria palustris* strain TAGA27	99.93	S1	St

TCA-11/OQ055140	*Qipengyuania aerophila* strain GH25	99.85	S2	Lig
TCA-150/OQ071775	*Streptomyces nigra* strain 452	100	S2	ASW
TCA-12/OQ055141	*Methylophaga pinxianii* strain TMB456	99.86	S2	St
TCA-13/OQ055142	*Methylophaga pinxianii* strain TMB457	98.74	S2	ASW
TCA-151/OQ071776	*Georgenia muralis* strain NBRC 103560	99.65	S2	Lig
TCA-152/OQ071777	*Georgenia muralis* strain NBRC 103560	99.79	S2	Lig
TCA-153/OQ071778	*Pseudorhizobium marinum* strain MGL06	99.71	S2	ASW
TCA-154 /OQ071779	*Halomonas titanicae* strain BH1	99.57	S2	Lig
TCA-155/OQ071780	*Pseudorhizobium marinum* strain MGL06	99.71	S2	Lig
TCA-156/OQ071781	*Stutzerimonas xanthomarina* strain KMM 1447	99.08	S2	Lig
TCA-157/OQ071782	*Aurantimonas coralicida* DSM 14790	99.85	S2	ASW
TCA-158/OQ071783	*Alcanivorax jadensis* strain T9	99	S2	St
TCA-22/OQ055151	*Colwellia asteriadis* strain KMD 002	98.84	S2	St
TCA-159/OQ071784	*Alloalcanivorax gelatiniphagus* strain MEBiC08158	99.86	S2	Lig
TCA-160/OQ071785	*Stutzerimonas stutzeri* strain CCUG 11256	99.79	S2	St
TCA-161/OQ071786	*Halalkalibacterium halodurans* strain DSM 497	100	S2	St
TCA-162/OQ071787	*Pseudoalteromonas atlantica* strain IAM 12927	99.78	S2	St
TCA-163/OQ071788	*Marinobacter shengliensis* strain SL013A34A2	99.71	S2	St
TCA-164/OQ071789	*Halomonas titanicae* strain BH1	99.51	S2	St
TCA-165/OQ071790	*Halomonas alkaliphila* strain 18bAG	99.37	S2	St

Table S3. Affiliation of potential novel isolates to various taxonomic groups and levels

Taxonomy at Phylum and class level		Cultivation method	Number of potential novel isolates by level			
			New species	New genus	New family	Total novel isolates recovered from different taxonomic classes
Pseudomonadota	*Alpha- proteobacteria*	DICA	29	23	3	55
		TCA	10	-	-	10
	Beta-proteobacteria	DICA	5	-	-	5
		TCA	-	-	-	-
	Gamma-proteobacteria	DICA	9	9	-	18
		TCA	6	-	-	6
Actinomycetota	*Acidimicrobiia*	DICA	4	3	-	7
		TCA	-		-	-
	Actinomycetia	DICA	12	-	-	12
		TCA	4	-	-	4
	Thermoleophilia	DICA	2	-	-	2
		TCA	-	-	-	-
Balneolota	*Balneolia*	DICA	2	-	-	2
		TCA	-	-	-	-
Cyanobacteria	*Cyanophyceae*	DICA	-	-	1	1
		TCA	-	-	-	-
Bacteroidota	*Cytophagia*	DICA	1	-	-	1
		TCA	-	-	-	-
	Flavobacteriia	DICA	8	-	-	8
		TCA	-	-	-	-
Verrucomicrobiota	*Opitutae*	DICA	-	4	-	4
		TCA	-	-	-	
Total number of novel isolates at species, genus and family level			92	39	4	135

Table S4. List of some of the previously uncultured bacteria reported in the studied samples, recovered by DICA

Uncultured bacteria reported in sediment samples	Previously uncultured strains reported in NCBI-NR database	Strains code/ Genebank accession number of isolates recovered
p_*Pseudomonadota*;c_*Alphaproteobacteria*;o_*Rhizobiales;*f__*Xanthobacteraceae;*g__*Pseudolabrys;*s_uncultured_bacterium	Uncultured bacterium clone WC3145 (GQ264048)	DICA-005/OQ055020<br>DICA-054/OQ055068<br>DICA-055/OQ055069<br>DICA-056/OQ055070
p_*Pseudomonadota*;c_*Gammaproteobacteria;*o__*Oceanospirillales*;f__*Alcanivoracaceae1;*g__*Alcanivorax;*s_uncultured_	Uncultured bacterium clone 16S22 (KX589511)	DICA-017/OQ055032<br>DICA-044/OQ055058<br>DICA-062/OQ055076<br>DICA-063/OQ055077
p_*Pseudomonadota;c_Alphaproteobacteria;*o_*Rhodospirillales;*f__*Magnetospiraceae;*g__*Magnetospira*;s_uncultured_marine	Uncultured bacterium clone NC55 (KJ590695)	DICA-083/OQ055097<br>DICA-087/OQ055101<br>DICA-090/OQ055104<br>DICA-093/OQ055107
p_*Pseudomonadota*;c_*Alphaproteobacteria;*o_uncultured;f_uncultured;g_uncultured;s_uncultured_Rhodobacteraceae	Uncultured bacterium clone: MPB2-25 (AB630701)	DICA-025/OQ055040
p_*Pseudomonadota*;c_*Alphaproteobacteria;*o__*Rhodobacterales;*f__*Rhodobacteraceae*;_;uncultured;__	Uncultured bacterium clone F10_10.2_1 (FJ716861.1)	DICA-009/OQ055024
p_*Actinomycetota;*c_*Acidimicrobiia;*o_*Actinomarinales;*f_uncultured;g__uncultured;__	Uncultured bacterium clone JS5_521 (KR825135)	DICA-021/OQ055036
p_*Actinomycetota;*c_*Acidimicrobiia;*o__*Microtrichales;*f_*Ilumatobacteraceae;*g__*Ilumatobacter;*s_uncultured_actinobacterium	Uncultured bacterium clone EzyYy191 (KX172283.1)	DICA-011/OQ055026<br>DICA-052/OQ055066<br>DICA-053/OQ055067
p_*Pseudomonadota*;c_*Gammaproteobacteria;*o_*Cellvibrionales*;f__*Porticoccaceae;*g__*Porticoccus;*s_uncultured_bacterium	Uncultured bacterium clone SIP6-6-11 (GU584713)	DICA-036/OQ055051
p_*Pseudomonadota*;c_*Alphaproteobacteria;*o__*Rhizobiales;*f__*Devosiaceae;*g__uncultured;__	Uncultured bacterium clone WW3_66 (GQ264514)	DICA-012/OQ055027
p_*Verrucomicrobiota;*c__*Verrucomicrobiae;*o__*Opitutales;*f_*Opitutaceae;*g__uncultured_bacterium;s__uncultured;	Uncultured bacterium clone 3C003229 (EU801852.1)	DICA-002/OQ055017<br>DICA-068/OQ055082<br>DICA-069/OQ055083

Table S5. Composition of each modified nutrient medium used in the study	
**Nutrient media**	**Composition**
0.5% Alkali Lignin (Lig-medium)	Prepared 5.0% stock solution of alkali-lignin by mixing with (10.0%) NaOH. 0.50 mL lignin solution mixed with 99.50 mL Artificial sea water
0.5% Starch (St-medium)	Prepared 5% stock solution of Starch by mixing with boiled-water. 0.5 mL starch solution mixed with 99.5 mL Artificial sea water
Artificial sea water (ASW-medium)	(g/l): NaCl 26.0 g: $MgCl_2 \cdot 6H_2O$ 5.0 g: $CaCl_2 \cdot 2H_2O$ 1.4 g: Na_2SO_4 4.0 g: NH_4Cl 0.3 g: KH_2PO_4 0.1 g: KCl 0.5 g, 1.0 mL trace element mixture, 30.0 mL 1.0 M $NaHCO_3$ solution, 1.0 mL vitamin mixture, 1.0 mL thiamine solution and 1.0 mL vitamin B_{12} solution.
Trace element mixture	HCl (25%) 13.0 mL: EDTA, disodium salt 5.2 g: H_3BO_3 10.0 mg: $MnCl_2.4H_2O$ 5.0 mg: $FeSO_4.7H_2O$ 2100.0 mg: $CoCl_2.6H_2O$ 190.0 mg: Nicl2.6H_2O 24.0 mg: $CuCl_2.2H_2O$ 10.0 mg: $ZnSO_4.7H_2O$ 144.0 mg: $Na_2MoO_4.2H_2O$ 36.0 mg: Distilled water 1000.0 mL: (pH 6.0 with NaOH): Autoclaved anoxically.
Vitamin mixture	Sodium phosphate (10.0 mM) 100.0 mL: 4-Aminobenzoic acid 4 mg: D(+) Biotin 1mg: Nicotinic acid 10.0 mg: D(+) Pantothenic acid, Calcium salt 5.0 mg and Pyridoxine dihydrochloride 15.0 mg.
Thiamine solution	Sodium phosphate (10.0 mM) 100.0 mL: Thiamine chloride dihydrochloride 10.0 mg.
Vitamin B_{12} solution	Distilled water 100.0 mL: Cyanocobalamin 5.0 mg.
50% diluted marine 2216E	g/l: Peptone, Yeast extract and Ammonium sulfate 0.25 g each: $FePo_4$ 0.005 g: Calcium carbonate 0.05 g: All these components were dissolved in 1-liter Artificial sea water. PH: 7.2-7.4: Agar: 1.5%
50% diluted marine R2A	g/l: Yeast extract, Tryptone, Casamino acid, Starch and Glucose 0.25 g each: Sodium pyruvate and Potassium di phosphate 0.15 g: Magnesium sulfate heptahydrate 0.025 g: All these components were dissolved in 1-liter Artificial sea water. pH: 7.2-7.4: Agar 1.5%
pH adjustments	0.5 M NaOH or 0.5 M HCl solutions were used to adjust pH of each medium.
Sterilization conditions	All media and mixtures were autoclaved for 20.0 min at 121.0 °C (15.0 psi) to excepting vitamins solutions which were filter-sterilized by (0.22 µm) pore size.
